# Identification and characterization of terpene synthase genes accounting for volatile terpene emissions in flowers of *Freesia* x *hybrida*

**DOI:** 10.1093/jxb/ery224

**Published:** 2018-06-12

**Authors:** Fengzhan Gao, Baofeng Liu, Min Li, Xiaoyan Gao, Qiang Fang, Chang Liu, Hui Ding, Li Wang, Xiang Gao

**Affiliations:** Key Laboratory of Molecular Epigenetics of MOE and Institute of Genetics & Cytology, Northeast Normal University, Changchun, China

**Keywords:** Emission pattern, fitness, flower scent, *Freesia*, ornamental plant, speciation, substrate selectivity, terpene synthase

## Abstract

The development of flower scents was a crucial event in biological evolution, providing olfactory signals by which plants can attract pollinators. In this study, bioinformatics, metabolomics, and biochemical and molecular methodologies were integrated to investigate the candidate genes involved in the biosynthesis of volatile components in two cultivars of *Freesia* x *hybrida*, Red River^®^ and Ambiance, which release different categories of compounds. We found that terpene synthase (*TPS*) genes were the pivotal genes determining spatiotemporal release of volatile compounds in both cultivars. Eight *FhTPS* genes were isolated and six were found to be functional: FhTPS1 was a single-product enzyme catalyzing the formation of linalool, whereas the other four FhTPS proteins were multi-product enzymes, among which FhTPS4, FhTPS6, and FhTPS7 could recognize geranyl diphosphate and farnesyl diphosphate simultaneously. The FhTPS enzymatic products closely matched the volatile terpenes emitted from flowers, and significant correlations were found between release of volatile terpenes and *FhTPS* gene expression. Graphical models based on these results are proposed that summarize the biosynthesis of *Freesia* floral volatile terpenes. The characterization of *FhTPS* genes paves the way to decipher their roles in the speciation and fitness of *Freesia*, and this knowledge could also be used to introduce or enhance scent in other plants.

## Teaser

The molecular and biochemical basis of spatiotemporal emissions of flower scent molecules in two *Freesia* cultivars is described, paving the way to investigate their roles in *Freesia* speciation and reproductive fitness.

## Introduction

The emergence of the specialized secondary metabolic pathways improved the adaptive ability of plants during their evolution (Waters, 2003). In particular, the widespread biosynthesis of volatile organic compounds (VOCs) in plant tissues has served multiple biological functions, including defense against pathogens, parasites, and herbivores (Holopainen and Gershenzon, 2010). The evolutionary emergence of angiosperms led to further exploitation of volatile compounds in flowers in order to attract pollinators (Filella *et al.*, 2013; Byers *et al.*, 2014). VOCs can be divided according to their independent origins into three categories, terpenes, benzenoid aromatics, and fatty acid derivatives (Dudareva *et al.*, 2013), among which terpenes with relatively low molecular weight (such as 10-carbon monoterpenes and 15-carbon sesquiterpenes) account for the largest proportion (Chen *et al.*, 2011; Dudareva *et al.*, 2013).

The metabolic pathways of volatile terpenes have been well characterized in the plant kingdom. Generally, the 5-carbon precursors isopentenyl diphosphate (IPP) and its allylic isomer dimethylallyl diphosphate (DMAPP) are generated and enter two independent pathways, the methylerythritol phosphate (MEP) pathway and the mevalonic acid (MVA) pathway, giving rise to the monoterpenes in plastids and the sesquiterpenes in cytosol (Vranová *et al.*, 2013; Kitaoka *et al.*, 2015). A series of enzymes participating in the catalytic reactions of volatile terpenes has been identified in both pathways. Among them, the terpene synthases (TPSs) are regarded as pivotal in the conversion of geranyl diphosphate (GPP) and farnesyl diphosphate (FPP) substrates into monoterpenes and sesquiterpenes, respectively (Degenhardt *et al.*, 2009; Chen *et al.*, 2011).

The TPSs are encoded by a gene family that is present in all angiosperm and gymnosperm genomes, and are phylogenetically classified into seven subfamilies (TPS-a to TPS-h) based on sequence relatedness and functional assessment (Chen *et al.*, 2011). To date, *TPS* genes have been extensively examined in many terrestrial plants, ranging from spermatophytes to mosses, but especially in core eudicot plants (Hayashi *et al.*, 2010; Falara *et al.*, 2011; Yahyaa *et al.*, 2015; Kumar *et al.*, 2016). *TPS* genes have been reported in Arabidopsis (Tholl and Lee, 2011), tomato (Falara *et al.*, 2011), orange (Dornelas and Mazzafera, 2007), eucalyptus (Külheim *et al.*, 2015), grape (Martin *et al.*, 2010), apple (Nieuwenhuizen *et al.*, 2013), and the basal angiosperm *Amborella* (Amborella Genome Project, 2013). Comparatively speaking, the majority of these *TPS* genes have been isolated from vegetative tissues and fruits in order to investigate their defensive roles. For instance, in maize, *TPS10* was induced in herbivore-damaged leaves and *TPS23* was responsible for attracting natural enemies of herbivores through controlling (*E*)-β-caryophyllene emissions (Köllner *et al.*, 2009; Capra *et al.*, 2015). However, their role in the biosynthesis of flower scents might be equally important from the perspective of plant evolution and speciation, as they perform crucial roles in attracting pollinators and, in combination with other floral traits, determine plant pollination syndromes (Ojeda *et al.*, 2013). For instance, it has been shown that variation of the ocimene synthase gene likely caused differential visitation of pollinators, to promote reproductive isolation between *Mimulus lewisii* and *Mimulus cardinalis* (Byers *et al.*, 2014). However, to our knowledge, previous efforts to identify the *TPS* genes from flowers have mainly been focused on dicotyledonous plants, such as *Osmanthus fragrans* (Zeng *et al.*, 2015), *Cananga odorata* (ylang ylang) (Jin *et al.*, 2015), *Laurus nobilis* (bay laurel) (Yahyaa *et al.*, 2015), *Matricaria recutita* (chamomile) (Irmisch *et al.*, 2012), and *Mimulus* (monkeyflowers) (Byers *et al.*, 2014), as well as *Arabidopsis thaliana* (Chen *et al.*, 2003; Tholl *et al.*, 2005). Only a few *TPS* genes have been isolated and functionally characterized from the flowers of monocot plants, such as *Hedychium coronarium* (Yue *et al.*, 2014) and *Alstroemeria* (Aros *et al.*, 2012). Given the importance of floral scent in evolution and speciation (Parachnowitsch *et al.*, 2012; Adler and Irwin, 2012), more *TPS* genes should be isolated from poorly studied clades of plants with diverse animal pollinators, especially petaloid monocots.

*Freesia* is a small genus of the Iridaceae, a large family (over 2000 species) that is well known for its colorful flowers and strong volatile scents (Goldblatt and Manning, 2006). Recently, three floral pollination syndromes were observed or predicted among the 16 wild species in this genus, involving pollination by (i) nectar-collecting bees, (ii) day-flying butterflies, and (iii) night-flying settling moths. Consistent with this pollinator diversity, different components and emission patterns of floral VOCs were detected in the genus, dominated by volatile terpenes and apocarotenoids. In addition, there is a clear phylogenetic pattern: phylogenetically closely related species have similar volatile compound profiles (Manning and Goldblatt, 2010). Therefore, it is plausible to deduce that volatile terpenes might be at least one of, or even the main, driving force for the reproductive isolation and speciation of *Freesia* species.

Because of the attractive scent of the flowers, the genus *Freesia* has a long history of cultivation in Europe, dating back to the late 18th century. The current cultivars, designated as *Freesia* x *hybrida*, are considered to be the results of crossing between *Freesia corymbosa* and *Freesia leichtlinii* (Wongchaochant *et al.*, 2005; Manning and Goldblatt, 2010), of which the latter is usually recognized as the most highly scented species in the genus. Unlike some other flowers, which lost their scent during breeding for visual appeal, most *Freesia* cultivars remain scented. In our previous studies, a large number of VOCs were detected in the flowers of one *Freesia* cultivar, Red River^®^, dominated by the monoterpene linalool, accompanied by minor amounts of sesquiterpenes (Fu *et al.*, 2007; Ao *et al.*, 2013). However, in contrast to the isolation and identification of the chemical components, no *TPS* genes have yet been functionally characterized in this plant.

In the present study, we selected two *Freesia* cultivars, Red River^®^ and Ambiance, with clearly distinct VOC profiles, to isolate the candidate *TPS* genes and decode their potential roles in the biosynthesis of volatile terpenes in the flowers. First, metabolomic and transcriptomic analyses were integrated (i) to confirm whether *TPS* genes had crucial roles in the differential biosynthesis of volatile terpenes in the two cultivars, and (ii) to determine the number of *TPS* genes functioning in the flowers of each cultivar. Second, the candidate *TPS* genes were subjected to biochemical analysis to verify their enzymatic products *in vitro*, and the major *TPS* genes were also characterized *in vivo* by overexpression in tobacco plants. Third, based on the correlation analysis of volatile terpene emission, spatiotemporal expression of *TPS* genes, enzymatic products, and substrate specificity of TPS proteins, diagrammatic models for the biosynthesis and release of volatile terpenes in Red River^®^ and Ambiance were proposed. We hope these results will provide new insights into terpene biosynthesis in monocot plants, as well as laying the foundation for deciphering their roles in the diversification and speciation within the genus *Freesia*. The *TPS* genes reported in this study also have potential applied significance, as *Freesia* scent is widely used in the manufacture of perfumes, scented oils and bathing products, and other similar personal and household products. Moreover, these genes could also be considered promising candidates for scent modification in other, unscented, horticultural plants.

## Materials and methods

### Plant materials and growth conditions

The *Freesia* x *hybrida* cultivars Red River^®^ and Ambiance were cultivated in a greenhouse with a photoperiod of 12 h, and with the temperature set at 25 °C in the light and 15 °C in the dark.

To analyze the natural volatile compounds, flowers from five different developmental stages were enclosed in a transparent device, which was made of inorganic materials, and then sampled. In addition, flowers at stage 5 were further divided into five tissues defined previously (Li *et al.*, 2016; Sun *et al.*, 2015; Sun *et al.*, 2016). Each tissue was sealed into solid-phase microextraction (SPME) vials immediately for further analysis.

To investigate the spatiotemporal correlation between the transcription profiles of *FhTPS* genes and the emission of volatile terpene compounds, a range of samples including five flower developmental stages and five flower tissues were collected for RNA extraction, as described in our earlier studies (Li *et al.*, 2016). All samples were immediately frozen in liquid nitrogen and stored at −80 °C until required. Wild-type plants of *Arabidopsis thaliana* (Columbia-0) used for subcellular localization in protoplasts were grown in a greenhouse at 22 °C with a photoperiod of 16 h light/8 h dark. Leaves of 3- to 4-week-old Arabidopsis plants were used for protoplast isolation. For *in vivo* activity assay of FhTPS proteins, tobacco plants were grown in a greenhouse at 22 °C under natural light. The youngest expanded leaves of 4-week-old tobacco plants were used for *Agrobacterium* infiltration experiments.

### Gas chromatography–mass spectrometry analysis of volatile compounds in flowers of *Freesia* x *hybrida*

Headspace SPME was employed to collect the volatile compounds from flower tissues, which were absorbed by a 75 μm CAR/PDMS fiber (Sigma-Aldrich) for 2 hours at 25 °C. Tetradecane (10 ng/ml) was added together with the samples as an internal standard. The fibers were stored at –20 °C before analysis by gas chromatography–mass spectrometry (GC-MS).

Total trapped volatile compounds were subsequently thermally desorbed and transferred to an Agilent 5975-6890N GC-MS apparatus (Agilent Technologies) equipped with a HP-1MS fused-silica capillary column (0.25 mm diameter, 30 m length, and 0.25 μm film thickness). The temperature program was isothermal at 60 °C for 3 min, then increased at a rate of 5 °C min^–1^ to 100 °C for 1 min , and was then further increased at a rate of 10 °C min^–1^ to 250 °C for 10 min. Compounds were identified by comparing mass spectra with the NIST 2008 mass spectra library as well as standard samples.

### DNA or RNA extraction and cDNA synthesis

In order to obtain the genomic sequences of the *FhTPS* genes, DNA was extracted from ﬂowers of Red River^®^ using the NuClean Plant Genomic DNA Kit (CWBIO) according to the manufacturer’s instructions. RNA was extracted from samples using the OminiPlant RNA Kit (DNase I) (CWBIO) following the manufacturer’s standard protocol. The purity and concentration of RNA were assessed using a Nanodrop 1000 spectrophotometer (Thermo Scientific, Waltham, MA, USA). cDNA was synthesized in a final reaction volume of 25 µl from total RNA (1 μg) using Oligo d(T)15 primers together with M-MLV Reverse Transcriptase (Promega) according to the manufacturer’s specifications.

### Gene cloning and sequence analysis

Homologous genes involved in the terpene biosynthetic pathway expressed in *Freesia* flowers were screened in a previously reported transcriptome database (Li *et al.*, 2016), by using the TBLASTN algorithm. Sequences obtained were subjected to a manual BLASTX search of National Center for Biotechnology Information (NCBI) data, and the best hits were taken as candidate genes. The SMARTTM RACE cDNA Amplification Kit (Clontech) was used to obtain complete open reading frames (ORFs) of *FhTPS* genes when necessary. Specific primers were then designed (see Supplementary Table S1 at *JXB* online) to amplify the full-length cDNA sequences. To ascertain the genomic structures of *FhTPS* genes, combinations of primers were designed and used in PCR reactions with genomic DNA as templates (Supplementary Table S1). PCR products of appropriate length were cloned into the *pGEM-T easy* vector (Promega) and then transformed into *Escherichia coli* JM109 competent cells before sequencing.

FhTPS proteins from *F. hybrida* were submitted to Clustal Omega to perform multiple sequence alignment. Conserved regions such as RRX_8_W, DDXXD, and NSE/DTE motifs were highlighted with different colors. The amino acid sequences of FhTPS proteins were analyzed with RaptorX and ChloroP to predict their three-dimensional structures (Källberg *et al.*, 2012) and subcellular locations (Emanuelsson *et al.*, 1999), respectively. For phylogenetic analysis, the full-length amino acid sequences of FhTPS proteins and their homologs in other plant species (see Supplementary Table S2) were aligned using Clustal Omega with default parameters (http://www.ebi.ac.uk/Tools/msa/clustalo/), and the alignments were analyzed using MEGA version 6 to generate a neighbor-joining tree with bootstrap analysis (1000 replicates) and gap handling using the Pairwise-Deletion option (Tamura *et al.*, 2013).

### Subcellular localization of FhTPS proteins

The intact ORF sequences of *FhTPS* genes were subcloned from the *pGEM-T easy* vector into the *pUC19* vector, which was driven by the constitutive *35S* cauliflower mosaic virus promoter, by replacing the termination codons with sequences encoding green fluorescent protein (GFP). The plasmids were then extracted using the GoldHi EndoFree Plasmid Maxi Kit (CWBIO) according to the manufacturer’s instructions. The constructs were transfected into protoplasts isolated from 3- to 4-week-old Arabidopsis rosette leaves before incubation at room temperature for 20–22 h in darkness, as described previously (Zhou *et al.*, 2014; Li *et al.*, 2016). Arabidopsis leaf protoplasts transiently expressing GFP and C-terminal GFP fusions of FhTPS proteins were visualized by fluorescence microscopy.

### Quantitative real-time PCR analysis

To investigate the expression profiles of *FhTPS* genes, a SYBR Green-based real-time PCR assay was carried out in a total volume of 10 μl of reaction mixture containing 5 μl of 2× Master Mix (TOYOBO), 0.5 μM of each primer, and 1 μl cDNA. The specific quantitative real-time PCR (qRT–PCR) primers of *FhTPS* genes are listed in Supplementary Table S1. The 18S rRNA gene was used as an internal control. Relative quantitative gene expression was calculated using the 2^−ΔΔCт^ formula (Livak and Schmittgen, 2001). All biological replicates were measured in triplicate.

### Heterologous expression of FhTPS proteins in *E. coli* and *in vitro* enzyme assay

To express FhTPS proteins in *E. coli*, the full-length sequences of *FhTPS* genes were amplified with specific primers (Supplementary Table S1) and then subcloned into the vector *pET-32a*. *FhTPS1*, *FhTPS2*, *FhTPS3*, *FhTPS4*, *FhTPS5*, and *FhTPS6* were amplified from the cultivar Red River^®^, whereas *FhTPS7* and *FhTPS8* were amplified from Ambiance. Subsequently, an empty vector and vectors harboring different *FhTPS* genes were used for transformation of *E. coli* strain BL21 (DE3). Recombinant proteins were induced by the application of 0.5 mM isopropyl-β-D-thiogalactopyranoside (IPTG); the optimal induction condition was 24 h and 16 °C. After induction, the cells were harvested by centrifugation, resuspended in phosphate-buffered saline, and disrupted by sonication. The crude proteins were then applied to a Ni Sepharose column (GE Healthcare). The purified proteins were collected and concentrated before enzyme assays. Unfortunately, FhTPS3, FhTPS4, and FhTPS5 failed to be induced as soluble proteins that collected into the supernatant, and thus the crude protein extracts were utilized in the following enzymatic activity assays.

The terpene synthase activity assays were conducted as described (Yahyaa *et al.*, 2015; Yue *et al.*, 2014) with some modifications. Briefly, the standard reaction mixture for the enzyme assay consisted of 25 mM HEPES as buffer (pH 7.4), 2 mM FPP (Sigma-Aldrich) or GPP (Sigma-Aldrich) as substrate, 15 mM MgCl_2_, 5 mM dithiothreitol, and 40–50 μg protein, in a total volume of 100 μl. The mixtures were incubated at 30 °C for 1 h, and meanwhile the volatile products were absorbed by a PDMS fiber before GC-MS analysis (as described above). For characterization of the kinetic parameters of FhTPS7, the assay was performed at 30 °C for 20 min with the standard reaction buffer described above. Reaction products were extracted in 50 µl of hexane, containing tetradecane as the internal standard, by immediate vigorous mixing, and 2 µl of the extract was used for GC-MS analysis. Values were determined using non-linear regression of the Michaelis–Menten equation. Extracts from *E. coli* transformed with *pET-32a* lacking the cDNA insert and heat-denatured FhTPS proteins served as controls and were run under the same conditions.

### *In vivo* characterization of FhTPSs

The intact *FhTPS* ORFs were cloned into the *pBI121* binary vector, and the obtained vectors were transformed into *Agrobacterium* cells (strain GV3101). The abaxial air spaces of the youngest leaves (>1 cm^2^) of 4-week-old tobacco plants were infiltrated with the *Agrobacterium* strains harboring *FhTPSs* together with a strain carrying the gene encoding the viral protein p19 (Nieuwenhuizen *et al.*, 2009; Green *et al.*, 2012). Freshly grown *Agrobacterium* cultures that reached an OD_600_ of 0.6–0.8 were centrifuged and resuspended in infiltration media [10 mM 2-(*N*-morpholino)ethanesulfonic acid, 10 mM MgCl_2_] and incubated without shaking at room temperature for 2–3 h. Before infiltration, cultures containing *FhTPS* genes or *p19* were mixed at a 1:1 ratio. After infiltration, tobacco plants were maintained in a growth chamber at 22 °C with 16 h light/8 h dark for 5 days. Infected leaves were collected and placed in 20 ml SPME vials for analysis of volatile compounds. The volatile terpene analysis was performed as described above. The leaves of tobacco infiltrated by *Agrobacterium* harboring the *p19* gene alone served as a negative control.

### Data analysis

Pearson correlation analysis between the expression levels of *FhTPSs* and related volatile compounds was done using SPSS Statistics software. Heatmap visualization was performed with HemI1.0.3.3 software. Data are presented as means ±SD.

## Results

### Volatile terpenes are emitted differentially from the flowers of two cultivars of *Freesia* x *hybrida*

The volatile compounds in flowers of two *Freesia* x *hybrida* varieties, Red River^®^ and Ambiance, were analyzed temporally and spatially by headspace SPME-GC-MS analysis. Generally speaking, the volatile compounds identified in *Freesia* flowers were mainly monoterpenes, sesquiterpenes, and carotenoid derivatives (apocarotenoids).

**Fig. 1.**
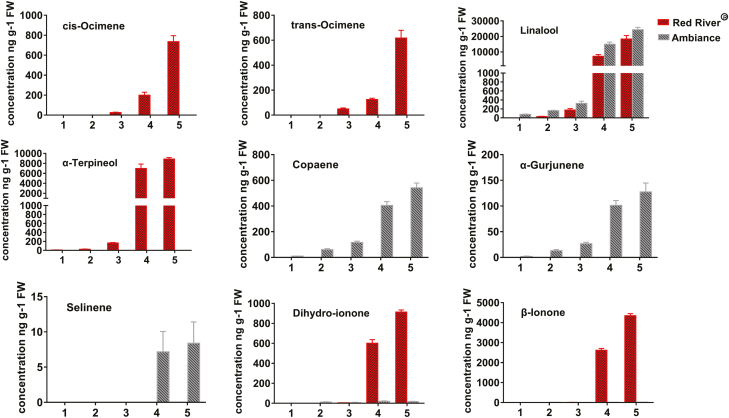
Representative volatile terpenes released during flower development of two cultivars of *Freesia* x *hybrida* (Red River^®^ and Ambiance). The terpenes were analyzed in five flower developmental stages, defined as in previous studies (Li *et al.*, 2016; Sun *et al.*, 2016; Sun *et al.*, 2016). The developmental stages of the two cultivars are shown in Supplementary Figs S1 and S2. Data are presented as mean ±SE, *n*=3.

In the course of development of *Freesia* flowers, the amount of volatile compounds increased gradually and peaked when the flowers opened fully, a finding consistent with human assessment of the floral scent (Fig. 1). A total of 31 terpenes were detected in *Freesia* flowers, 19 from Red River^®^ and 20 from Ambiance (Supplementary Table S3). Linalool was the most abundant terpene compound detected in flowers of both cultivars, accounting for 52.5% in Red River^®^ and 93.22% in Ambiance at Stage 5 (Supplementary Table S3). However, other volatile terpene compounds showed differing emission profiles in flowers of the two cultivars. α-Pinene, β-pinene, 1,8 cineole, D-limonene, *cis*-ocimene, *trans*-ocimene, terpinolene, (–)-4-terpineol, α-terpineol, nerolidol, and α-cyclocitral could be detected only in flowers of Red River^®^, whereas flowers of Ambiance were able to produce hotrienol, copaene, elemene, α-gurjunene, caryophyllene, α-guaiene, α-patchoulene, sativene, γ-cadinene, selinene, α-bulnesene, and vatirenene, which were not detected in Red River^®^ (Supplementary Table S3). Generally, flowers of Red River^®^ released more monoterpenes, mainly composed of α-terpineol (25.4%), *cis*-ocimene (2.1%), *trans*-ocimene (1.8%), and 1,8 cineole (0.9%), while copaene (2.5%) and α-gurjunene (0.6%) contributed most to the higher abundance of sesquiterpenes in the flowers of Ambiance (Supplementary Table S3).

**Fig. 2.**
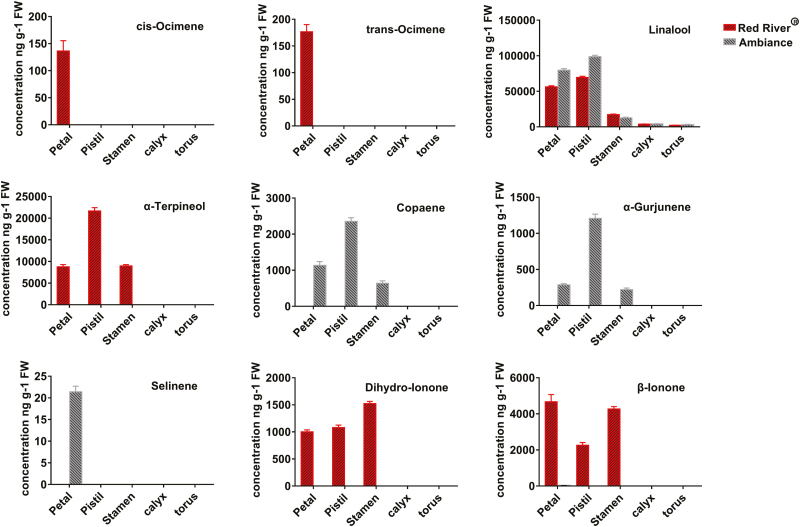
Representative volatile terpenes released from five flower tissues of two cultivars of *Freesia* x *hybrida* (Red River^®^ and Ambiance). The terpenes were analyzed in torus, calyx, petal, stamen, and pistil. The five flower tissues of the two cultivars are shown in Supplementary Figs S1 and S2. Data are presented as mean ±SE, *n*=3.

In order to further investigate the spatial release patterns of the volatile terpenes, flowers at Stage 5 were further divided into five tissues, the torus, calyx, petal, stamen, and pistil (Supplementary Figs S1 and S2). Interestingly, linalool was still the most predominant volatile terpene in different floral tissues (Fig. 2) and the unique component in calyces and toruses. Large concentrations of volatiles were released from petals and also from pistils and stamens (Fig. 2). More detailed data are provided in Supplementary Table S4.

### *FhTPS* genes play predominant roles in the differential emissions of volatile terpenes between the two *Freesia* cultivars

**Fig. 3.**
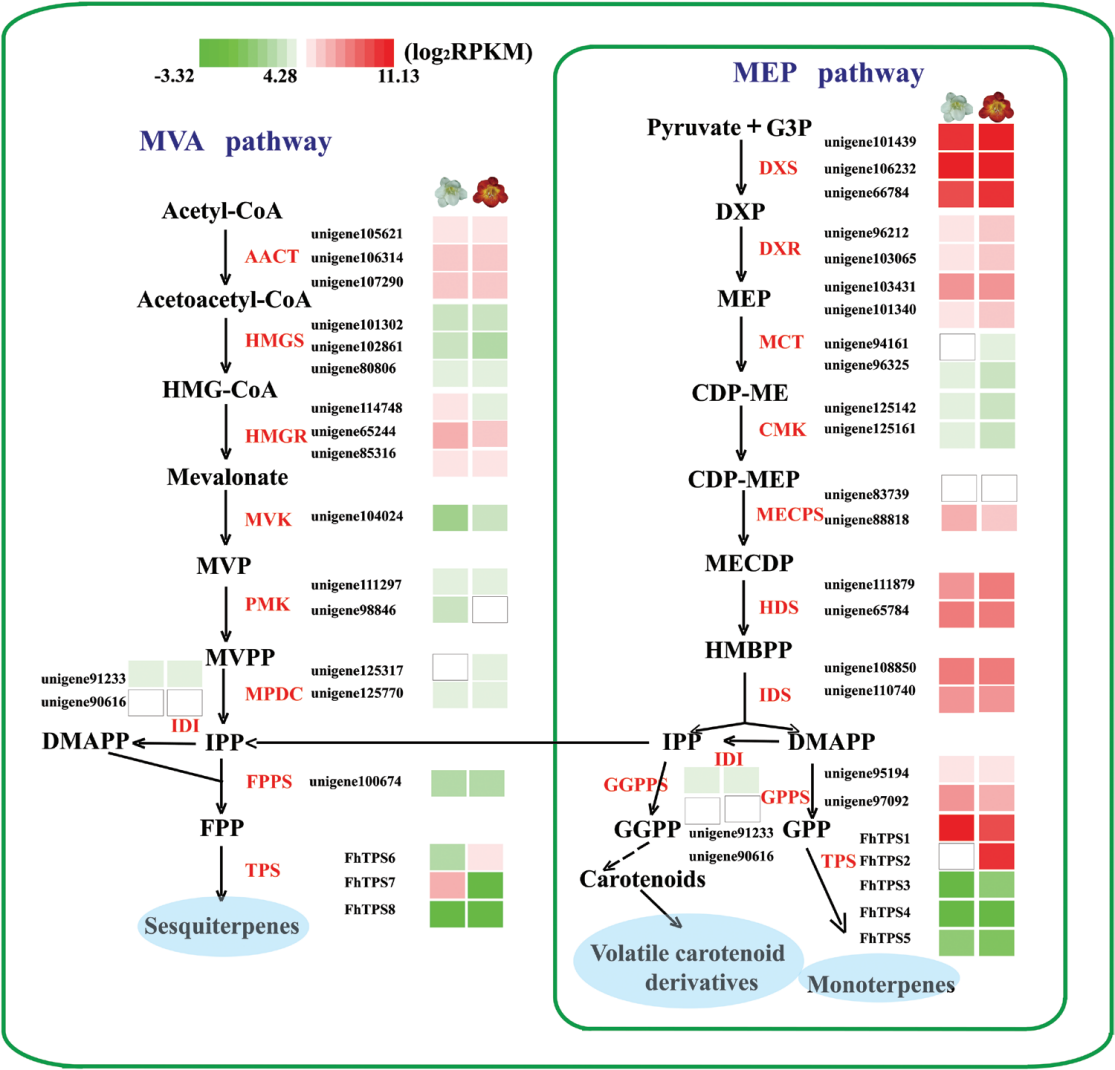
Expression pattern of genes in the MEP and MVA pathways of two *Freesia* cultivars. Gene expression levels in the fully opened flowers of Red River^®^ (red flower) and Ambiance (white flower) are represented by color gradations. The comparison and assignment of the TPSs in the MEP and MVA pathways is based on homology alignment. AACT, acetyl-CoA acetyltransferase; CMK, 4-(cytidine 59-diphospho)-2-C-methyl-D-erythritol kinase; DXR, 1-deoxy-D-xylulose 5-phosphate reductoisomerase; DXS, DXP synthase; FPPS, farnesyl diphosphate synthase; GPPS, geranyl diphosphate synthase; HDS, 4-hydroxy-3-methylbut-2-en-1-yl diphosphate synthase; HMGR, HMG-CoA reductase; HMGS, HMG-CoA synthase; IDI, isopentenyl diphosphate isomerase; IDS, isoprenyl diphosphate synthase; MCT, 2-C-methyl-D-erythritol 4-phosphate cytidylyltransferase; MECPS, ME-CDP synthase; MPDC, mevalonate diphosphate decarboxylase; MVK, mevalonate kinase; PMK, phosphomevalonate kinase; TPS, terpene synthase.

In plants, volatile terpenes originate from two distinct pathways, designated the MEP and the MVA pathway. In the present study, the structural genes encoding enzymes involved in both pathways were isolated, their expression levels in flowers at Stage 5 were evaluated and compared between the two cultivars (Fig. 3), and 46 candidate genes were identified. The candidate genes involved in the MEP pathway had relatively higher expression levels than the candidate genes of the MVA pathway, which was in accordance with the larger amounts of monoterpenes detected in both cultivars. In addition, almost all the candidate genes had similar expression levels between Red River^®^ and Ambiance except several *TPS* genes (nominated as *FhTPS* genes). Consequently, it was reasonable to speculate that the differentially expressed *FhTPS* genes might produce the divergent volatile compound emissions from the flowers of the two *Freesia* cultivars.

Based on their floral expression levels, putative *FhTPS* genes were further amplified. A total of eight *FhTPS* genes were obtained, designated *FhTPS1*–*FhTPS8*. All eight *FhTPS* genes were amplified from Red River^®^ except *FhTPS7*, and they were all also obtained from Ambiance with the exception of *FhTPS5*. Several variations were found between FhTPS proteins from the two cultivars, although FhTPS1 showed identical amino acid sequences (Supplementary Figs S3–S8). As shown in Supplementary Table S5, the *FhTPS1*–*FhTPS8* ORF sequences encoded 592, 595, 590, 566, 607, 566, 570, and 566 deduced amino acids, respectively, and showed high sequence identities with TPS proteins from other species. In order to obtain the genomic sequences of the eight *FhTPS* genes, specific primers (Supplementary Table S1) were designed and amplified using genomic DNA of Red River^®^ and Ambiance. Results showed that *FhTPS2*, *FhTPS3, FhTPS4*, and *FhTPS8* contained six introns, *FhTPS6* contained three introns, in both cultivars. In contrast, *FhTPS1* was found to have three introns in Red River^®^, whereas no intron was present in the corresponding gene in Ambiance. Furthermore, the genome sequence of *FhTPS5*, which had three introns in Red River^®^, could not be isolated from Ambiance, and *FhTPS7* was obtained only from Ambiance, in which it had six introns (Supplementary Fig. S9).

### FhTPSs phylogenetically cluster into different subgroups with different amino acid motifs and divergent subcellular localization

Sequence alignment revealed that all of the FhTPS proteins contained the conserved DDXX(D/E) and (N,D)DX2(S,T,G)X3E (NSE/DTE) regions that are essential for the binding of Mg^2+^ or Mn^2+^ cofactors to catalyze terpene biosynthesis (Supplementary Fig. S10). Moreover, FhTPS1, FhTPS2, FhTPS3, and FhTPS5 also shared another conserved motif, RRX_8_W, which is usually found in TPSs catalyzing the cyclization of monoterpenes.

**Fig. 4.**
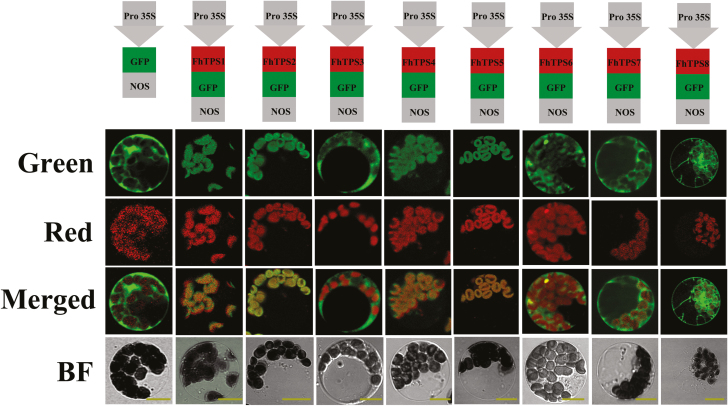
Subcellular localization of free GFP and eight FhTPS-GFP fusions in Arabidopsis leaf protoplasts. Green, GFP fluorescence detected in the green channel; Red, chlorophyll autofluorescence detected in the red channel; Merged, merged green and red channel images; BF, brightfield image. Bars=25 μm.

The biosynthesis of monoterpenes and sesquiterpenes is thought to be compartmentalized, with monoterpenes produced in the plastids, where GPP is synthesized, and sesquiterpenes formed in the cytosol, where FPP is generated (Chen *et al.*, 2011; Dudareva *et al.*, 2013). Bioinformatic analysis using the online ChloroP 1.1 (http://www.cbs.dtu.dk/services/ChloroP/) and RaptorX (Källberg *et al.*, 2012) software predicted that FhTPS1, FhTPS2, FhTPS4, and FhTPS5 had transit peptides positioned upstream of the RRX_8_W motif and therefore had a high probability of localizing in the plastids (Supplementary Fig. S11). This is consistent with the subcellular localization analysis, which showed that FhTPS1, FhTPS2, FhTPS4, and FhTPS5 were localized to the plastids, whereas FhTPS3, FhTPS6, FhTPS7, and FhTPS8 showed a diffuse cellular distribution (Fig. 4).

**Fig. 5.**
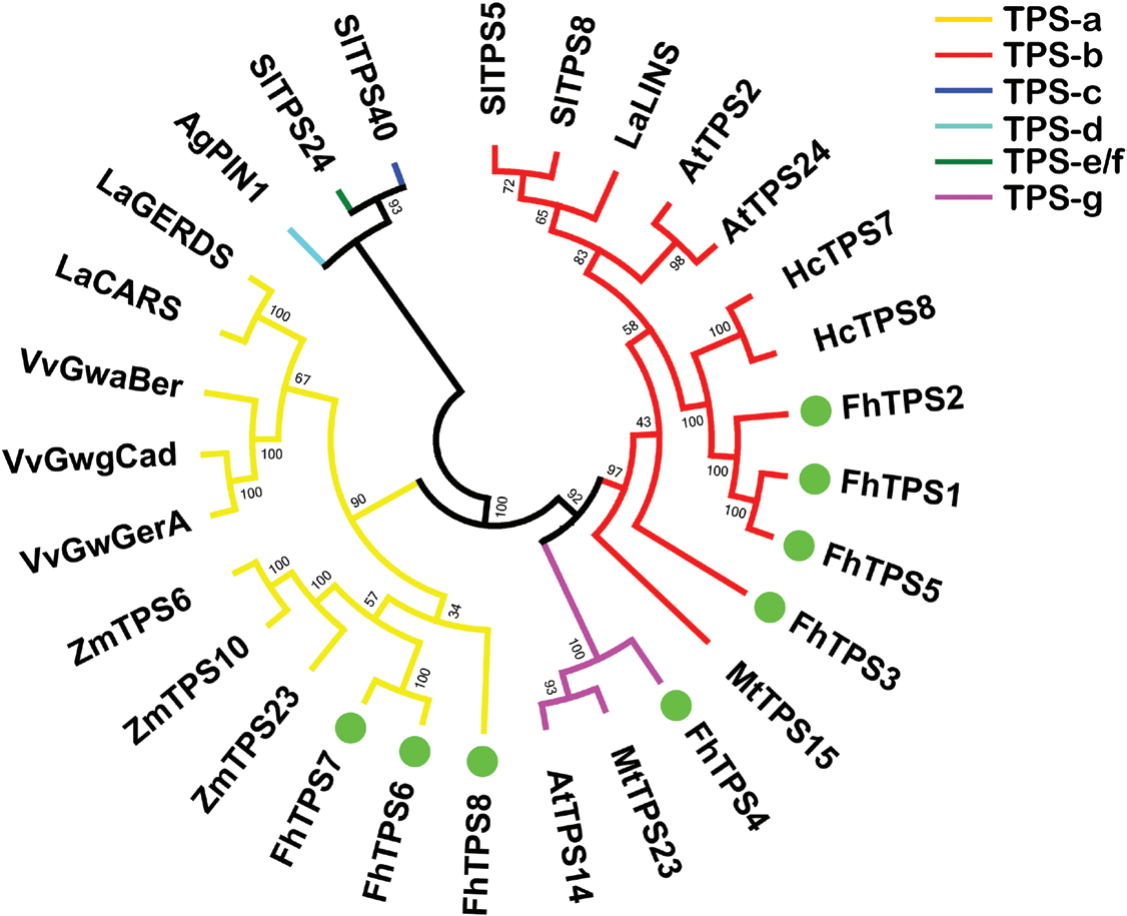
Results of phylogenetic analysis of TPS proteins from *Freesia* x *hybrida* (FhTPS1–FhTPS8) and other plants, done using the neighbor-joining method by MEGA6 software. Bootstrap values are shown as a percentage of 1000 replicates. *Freesia* TPS proteins in this study are highlighted by circles. The TPS-a, TPS-b, TPS-g, TPS-d, TPS-e/f, and TPS-c clades are highlighted with different shaded lines. Plant species are as follows: *Arabidopsis thaliana* (At); *Solanum lycopersicum* (Sl); *Abies grandis* (Ag); *Medicago truncatula* (Mt); *Zea mays* (Zm); *Hedychium coronarium* (Hc); *Vitis vinifera* (Vv); *Lavandula angustifolia* (La). Detailed information on the TPS proteins is provided in Supplementary Table S3.

To further clarify the potential roles of the eight FhTPS proteins, a phylogenetic tree was generated by the neighbor-joining method. The results showed that TPS proteins from various species were clearly classified into six different clades, including clades TPS-c (most conserved among land plants), TPS-e/f (conserved among vascular plants), and TPS-d (gymnosperm specific), and three angiosperm-specific clades, TPS-b, TPS-g, and TPS-a (Fig. 5). The TPS-a clade was further divided into a dicot-specific subclade and a monocot-specific subclade. All the FhTPS proteins identified in the present study clustered into angiosperm-specific clades. In particular, FhTPS6, FhTPS7, and FhTPS8 clustered into the TPS-a monocot subclade together with other TPS proteins from monocot plant species. Other FhTPS proteins, including FhTPS1, FhTPS2, FhTPS3, and FhTPS5, fell into the TPS-b clade. By contrast, FhTPS4 clustered independently into the TPS-g clade.

### *FhTPS* genes show different expression patterns during ﬂower development and in different tissues

**Fig. 6.**
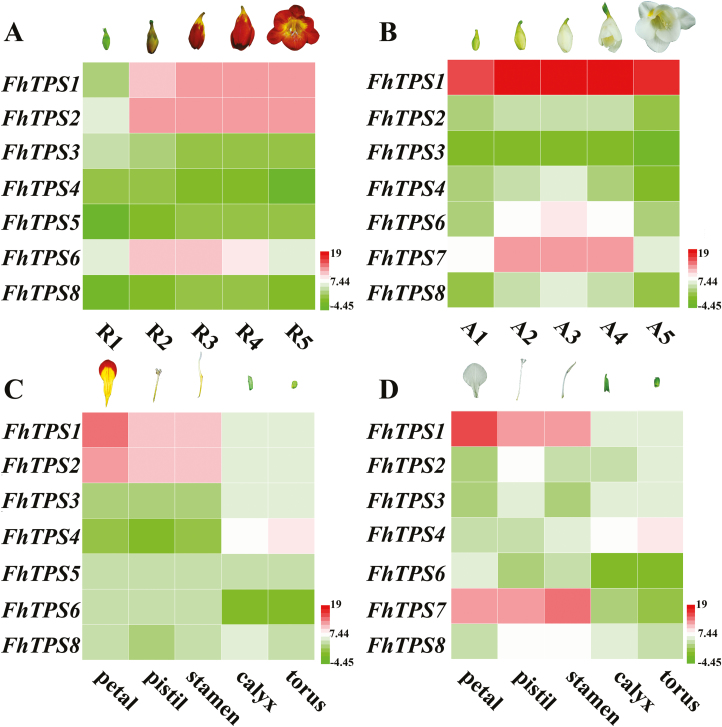
Spatiotemporal expression patterns of *FhTPS* genes in the flowers of Red River^®^ and Ambiance. (A) Expression levels of *FhTPS* genes in Red River^®^ flowers at different developmental stages (labelled R1–R5). (B) Expression levels of *FhTPS* genes in Ambiance flowers at different developmental stages (labelled A1–A5). (C) Expression levels of *FhTPS* genes in five flower tissues of Red River^®^. (D) Expression levels of *FhTPS* genes in five flower tissues of Ambiance. Gene expression levels (log_2_^2−ΔΔCт^) are represented by color gradations, as defined in the lower right of each panel. The flower developmental stages and tissues were defined as in previous studies (Li *et al.*, 2016; Sun *et al.*, 2016; Sun *et al.*, 2016). All results are presented as the means ±SD of triplicate experiments.

To compare the transcription of the *FhTPS* genes with the patterns of volatile terpene release during ﬂower development, floral development was divided into five stages as described in our previous studies (Sun *et al.*, 2015, 2016; Li, *et al.*, 2016). qRT-PCR was performed to investigate the temporal pattern of expression levels of the eight candidate *FhTPS* genes during flower development. In agreement with the patterns of volatile terpene emission, the transcript levels of most of the *FhTPS* genes substantially increased and were maintained at a high level during anthesis. In addition, *FhTPS1*, *FhTPS2*, and *FhTPS6* were highly expressed in Red River^®^ (Fig. 6A; Supplementary Fig. S12), whereas *FhTPS1* and *FhTPS7* had higher expression levels in Ambiance (Fig. 6B; Supplementary Fig. S12), implying specific functions in each *Freesia* cultivar.

To further investigate whether the expression patterns of *FhTPS* genes coincided spatially with terpene emissions, flower tissue-specific expression patterns were also assessed (Fig. 6C, D; Supplementary Fig. S13). qRT-PCR analysis showed that the expression level of *FhTPS1* and *FhTPS2* in Red River^®^, and *FhTPS1* and *FhTPS7* in Ambiance, were significantly higher than the other *FhTPS* genes in all the tested tissues. Furthermore, it was noteworthy that only *FhTPS4* showed a relatively high expression level in calyx and torus, indicating that it might be responsible for the biosynthesis and emission of terpenes in these two flower tissues. However, more detailed data are needed in order to decipher the catalytic properties of the FhTPS proteins. In addition, we found that the 18S rRNA could be used as a suitable endogenous control for qRT-PCR analysis as it is stably expressed (Supplementary Fig. S14).

### Biochemical characterization of the enzymes encoded by *FhTPS* genes revealed their versatile and diverse functions

The predominant volatile terpenes in most flowers are monoterpenes and sesquiterpenes, produced by pathways catalyzed by TPS proteins using GPP or FPP as substrate, respectively. To further confirm the enzymatic properties of the FhTPS proteins and their dominant roles in the biosynthesis of terpenes in *F. hybrida*, substrate specificity analyses were conducted using both GPP and FPP. To prepare recombinant proteins for the biochemical analysis, the eight *FhTPS* genes were expressed in *E. coli*. FhTPS1, FhTPS2, FhTPS6, FhTPS7, and FhTPS8 were induced into the supernatant and then purified as homogenous soluble proteins, whereas FhTPS3, FhTPS4, and FhTPS5 were expressed as insoluble inclusion bodies, and so crude protein extracts were utilized in the assays of enzymatic activity (Supplementary Fig. S15); although some FhTPS4 was detected in the supernatant, it failed to be purified. As expected, no products were detected when heat-inactivated recombinant proteins were added to reaction mixtures supplemented with both substrates. Therefore, only the crude protein extracts from the *E. coli* expression system containing empty vector were used as controls in the biochemical assays.

As shown in Table 1 and Supplementary Fig. S16, upon incubation with GPP as a substrate, both FhTPS1 and FhTPS4 exclusively catalyzed the formation of linalool, as predicted above, whereas FhTPS2, FhTPS6, and FhTPS7 were confirmed to be versatile enzymes with multiple products. Specifically, FhTPS2 mainly converted GPP into α-terpineol (78.7%) and a few other monoterpenes, such as 1.8 cineole (6.9%), D-limonene (3.9%), α-pinene (3.2%), myrcene (1.6%), and bicyclo[3.1.0]thujene (1.2%). FhTPS6 primarily catalyzed the formation of myrcene (40.1%), *cis*-ocimene (22.4%), *trans*-ocimene (16.1%), D-limonene (6.6%), linalool (4.9%), terpinolene (3.1%), terpinene (2.6%), isoterpinolene (2.2%), and thujene (2.1%). Similar to FhTPS6, FhTPS7 chiefly transformed GPP into the same monoterpenes with variable percentages, mainly myrcene (41.9%), *cis*-ocimene (21.3%), and *trans*-ocimene (16.6%). In contrast, no monoterpene was detected in assays using FhTPS3, FhTPS5, or FhTPS8 as enzymes.

**Table 1.** Enzymatic products catalyzed by FhTPS proteins

Enzymatic products	FhTPS1		FhTPS2		FhTPS4		FhTPS6		FhTPS7		FhTPS8	
	GPP	FPP	GPP	FPP	GPP	FPP	GPP	FPP	GPP	FPP	GPP	FPP
**Monoterpene**												
Bicyclo[3.1.0]Thujene	–	–	1.2%	–	–	–	–	–	–	–	–	–
α-Pinene	–	–	3.2%	–	–	–	–	–	–	–	–	–
Myrcene	–	–	1.6%	–	–	–	40.1%	–	41.9%	–	–	–
Thujene	–	–	–	–	–	–	2.1%	–	1.4%	–	–	–
Isoterpinolene	–	–	–	–	–	–	2.2%	–	2.2%	–	–	–
D-Limonene	–	–	3.9%	–	–	–	6.6%	–	6.2%	–	–	–
1,8 Cineole	–	–	6.9%	–	–	–	–	–	–	–	–	–
*trans*-Ocimene	–	–	–	–	–	–	16.1%	–	16.6%	–	–	–
*cis*-Ocimene	–	–	–	–	–	–	22.4%	–	21.3%	–	–	–
Terpinene	–	–	–	–	–	–	2.6%	–	1.4%	–	–	–
Terpinolene	–	–	–	–	–	–	3.1%	–	3.3%	–	–	–
Linalool	100%	–	–	–	100%	–	4.9%	–	5.6%	–	–	–
α-Terpineol	–	–	78.7%	–	–	–	–	–	–	–	–	–
**Sesquiterpene**												
α-Cubebene	–	–	–	–	–	–	–	1.5%	–	1.5%	–	–
Cycloisosativene	–	–	–	–	–	–	–	–	–	0.3%	–	–
Copaene	–	–	–	–	–	–	–	5.9%	–	37.1%	–	–
Epi-bicyclosesquiphellandrene	–	–	–	–	–	–	–	–	–	2.3%	–	–
Elemene	–	–	–	–	–	–	–	18.7%	–	11.1%	–	–
β-Maaliene	–	–	–	–	–	–	–	–	–	9.4%	–	–
β-Caryophyllene	–	–	–	–	–	–	–	–	–	0.6%	–	–
α-Guaiene	–	–	–	–	–	–	–	–	–	1.1%	–	–
Farnesene	–	–	–	–	–	–	–	–	–	0.4%	–	5.0%
β-Cubebene	–	–	–	–	–	–	–	–	–	–	–	1.6%
γ-Maaliene	–	–	–	–	–	–	–	–	–	1.5%	–	–
Selinene	–	–	–	–	–	–	–	66.5%	–	–	–	2.7%
Isoledene	–	–	–	–	–	–	–	–	–	–	–	1.2%
Unknown	–	–	–	–	–	–	–	–	–	–	–	0.9%
Caryophyllene	–	–	–	–	–	–	–	3.9%	–	0.4%	–	–
Acoradien	–	–	–	–	–	–	–	–	–	–	–	0.9%
Sativene	–	–	–	–	–	–	–	–	–	3.2%	–	–
α-Gurjunene	–	–	–	–	–	–	–	–	–	4.4%	–	46.9%
γ-Cadinene	–	–	–	–	–	–	–	–	–	1.7%	–	–
γ-Muurolene	–	–	–	–	–	–	–	–	–	0.8%	–	–
Chamigrene	–	–	–	–	–	–	–	–	–	–	–	11.0%
γ-Gurjunene	–	–	–	–	–	–	–	–	–	2.9%	–	–
Zingiberene	–	–	–	–	–	–	–	–	–	–	–	1.0%
Germacrene	–	–	–	–	–	–	–	–	–	2.0%	–	2.2%
α-Muurolene	–	–	–	–	–	–	–	–	–	2.9%	–	–
Guaia-1(10),11-diene	–	–	–	–	–	–	–	–	–	2.4%	–	4.0%
Eudesmene	–	–	–	–	–	–	–	–	–	1.4%	–	–
Nerolidol	–	–	–	–	–	100%	–	3.4%	–	2.1%	–	16.9%
Sesquiphellandrene	–	–	–	–	–	–	–	–	–	–	–	1.6%
Cadina-3,9-diene	–	–	–	–	–	–	–	–	–	6.7%	–	2.3%
1R,3Z,9S-2,6,10,10-Tetramethylbicyclo[7.2.0] undeca-2,6-diene	–	–	–	–	–	–	–	–	–	1.7%	–	–
Naphthalene,1,2,3,4,4a,7-hexahydro-1,6-dimethyl-4-(1- methylethy)	–	–	–	–	–	–	–	–	–	1.9%	–	1.8%

FPP, Farnesyl diphosphate; GFP, geranyl diphosphate;–, Not detected.

Catalytic activity analysis of the eight FhTPS proteins was also performed using FPP as substrate (Table 1; Supplementary Fig. S17). Results showed that FhTPS1, FhTPS2, FhTPS3, and FhTPS5 did not have the ability to synthesize sesquiterpenes, whereas FhTPS4, FhTPS6, FhTPS7, and FhTPS8 had versatile roles in the biosynthesis of sesquiterpenes. Notably, GC-MS analysis of the products of the reactions catalyzed by FhTPS7 identified at least 24 kinds of sesquiterpenes, with copaene (37.1%), elemene (11.1%), β-maaliene (9.44%), α-gurjurene (4.4%), sativene (3.2%), α-muurolene (2.9%), γ-gurjunene (2.9%), guaia-1(10),11-diene (2.4%), γ-cadinene (1.7%), and cycloisosativene (0.33%) as the major products. FhTPS8 and FhTPS6 were also shown to be multiple-product sesquiterpene synthases, which mainly catalyzed the formation of α-gurjunene and selinene, respectively, together with other sesquiterpenes. For FhTPS4, only nerolidol could be identified as a sesquiterpene product.

### Functional characterization of major *FhTPS* genes *in planta* was consistent with production obtained *in vitro*

**Fig. 7.**
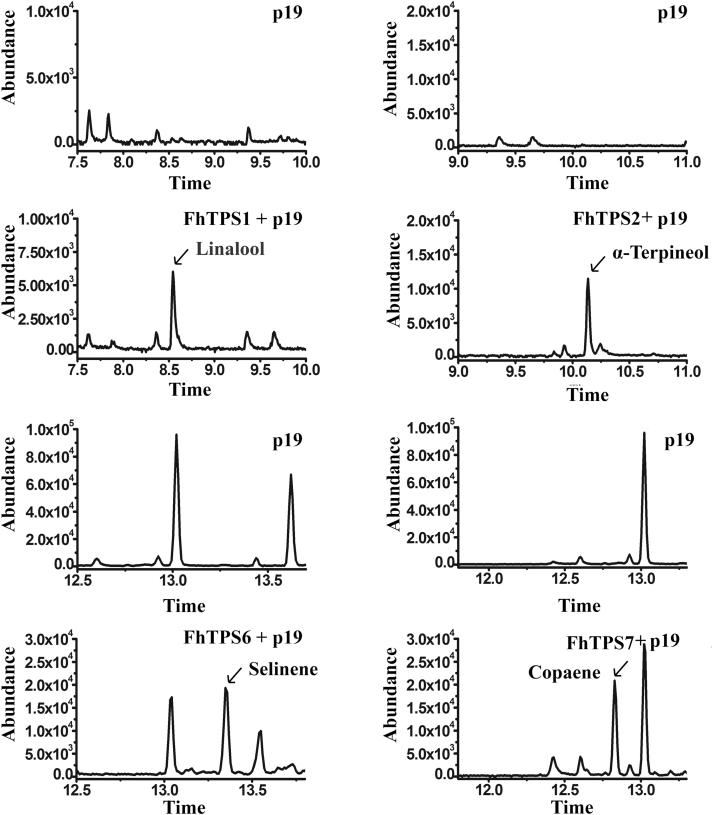
*In vivo* characterization of *FhTPS* genes. *FhTPS* genes together with *p19* were transiently expressed in tobacco leaves by *Agrobacterium*-mediated infiltration; the corresponding ectopic proteins could use the tobacco endogenous substrate to generate volatile terpenes. The products produced in tobacco leaves on the fifth day after transformation were analyzed by GC-MS. New product peaks were observed in contrast to controls and were identified by comparing mass spectra with the NIST 2008 mass spectra library. The mass spectra of products, e.g. linalool and α-terpineol, are shown in Supplementary Fig. S19. Leaves of tobacco infiltrated by *p19* alone were used as a control. The X-axis represents the retention time of the peak outflow, and the Y-axis represents the integrated area of the chromatographic peak.

To further investigate whether the major *FhTPSs* that are highly expressed in flowers of the two *Freesia* cultivars yield the same terpene products *in vivo*, they were transiently expressed in tobacco leaves. The major products detected in the transgenic tobacco leaves *in planta* were well matched with those detected in the previous biochemical analysis *in vitro*. Specifically, FhTPS1 could catalyze the formation of linalool when overexpressed, whereas transformation with *FhTPS2* caused the significant production of α-terpineol. For the sesquiterpene synthases, FhTPS6 and FhTPS7, the major components of the enzymatic products *in vitro*, that is, selinene and copaene, respectively, were detected in the transgenic tobacco leaves (Fig. 7). We also transformed *FhTPS3* and *FhTPS5* into tobacco; consistent with the biochemical analysis, no products were found to be synthesized or highly increased (data not shown), indicating that these two *FhTPS* genes might be pseudofunctional or that their catalytic activities were too low to yield detectable products.

### Enzyme kinetic parameter analysis demonstrates that FPP is the preferred substrate for FhTPS7 in the cultivar Ambiance

Regardless of linalool, more sesquiterpenes were released from flowers of Ambiance than from Red River^®^, and this might be ascribed to the higher expression of *FhTPS7* in Ambiance. However, biochemical analysis showed that FhTPS7 could synthesize monoterpenes using GPP and generate sesquiterpenes in the presence of FPP. Therefore, it was reasonable to deduce that the sesquiterpene production in Ambiance might be regulated by substrate selectivity of FhTPS7. In order to investigate this possible substrate bias, kinetic parameters of FhTPS7 were examined. A range of concentrations of GPP (3–270 μM) and FPP (1–135 μM) was employed to yield hyperbolic saturation curves; the results indicated that the recombinant FhTPS7 enzyme recognized FPP more efficiently (nearly 40-fold difference of *k*_cat_/*K*_m_) (Table 2), which is in accordance with the expectations suggested above. Therefore, it may reasonably be concluded that the substrate preference of FhTPS7 protein plays an important role in determining the abundance of sesquiterpenes in Ambiance.

**Table 2.** Kinetic parameters toward farnesyl diphosphate (FPP) and geranyl diphosphate (GPP) for recombinant FhTPS7

Substrate	*K*_m_ (μM)	*k*_cat_ (s^–1^)	*k*_cat_/*K*_m_ (s^–1^ M^–1^)
GPP	3.71 ± 0.19	1.46 × 10^–5^±3.07 × 10^–7^	3.94 ± 0.08
FPP	5.01 ± 0.21	7.32 × 10^–4^±1.25 × 10^–5^	146.41 ± 2.49

All results are presented as the mean ±SD of triplicate experiments.

### Expression of *FhTPS* genes is associated with the formation of volatile terpenes in the two cultivars of *Freesia* x *hybrida*

**Fig. 8.**
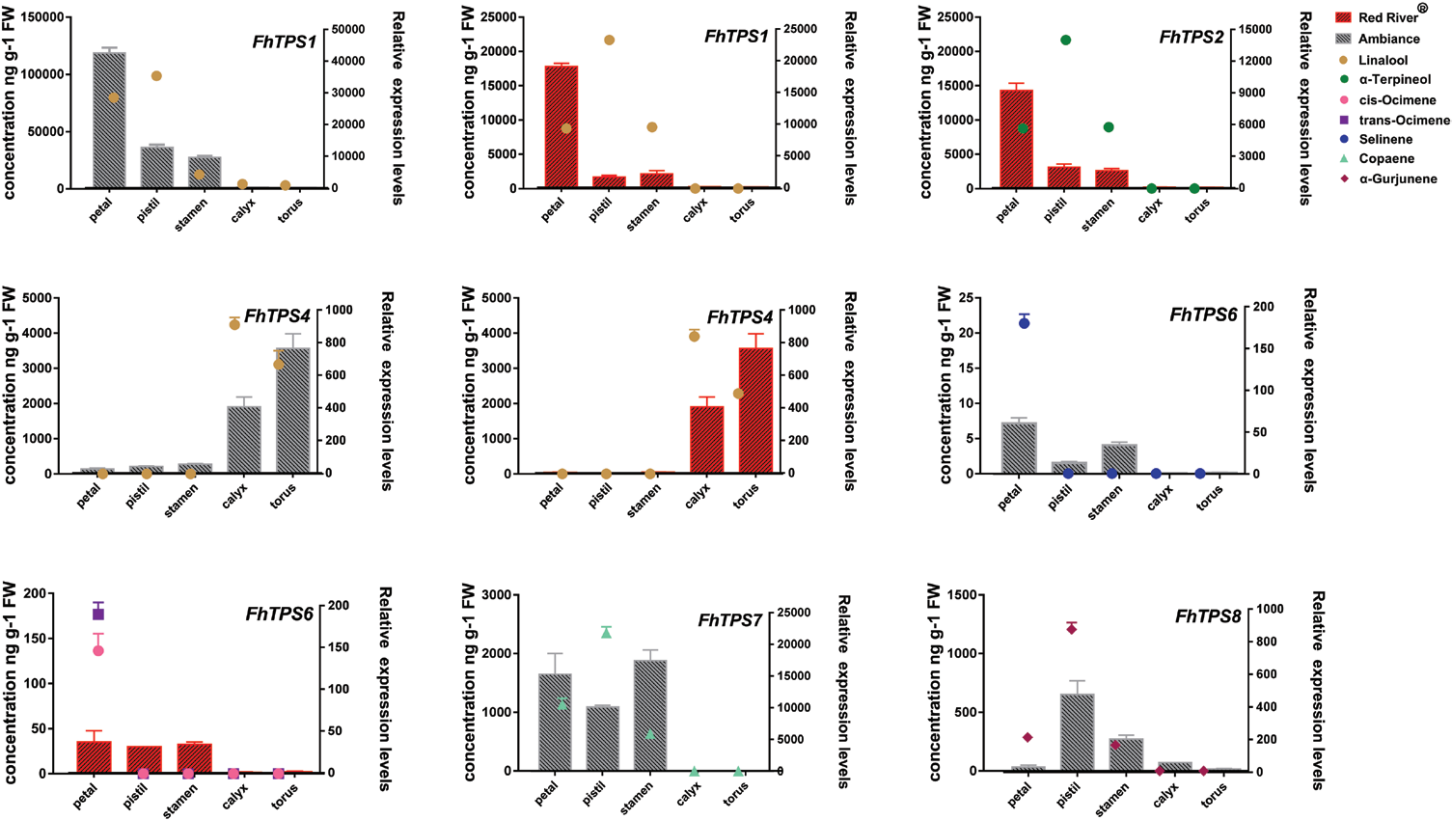
Correlation analysis between *FhTPS* gene expression patterns and volatile terpene emissions in different flower tissues of the two *Freesia* cultivars. The relative expression pattern of the major *FhTPS* genes (*FhTPS1*, *FhTPS2*, *FhTPS4*, *FhTPS6*, *FhTPS7*, and *FhTPS8*) was positively correlated with the concentration of the related volatile compound in different flower tissues. The left Y-axis represents the concentration of volatile compound; the right Y-axis represents the relative expression levels of the *FhTPS* genes. Data are presented as means ±SD.

As the major volatile terpene components released from flowers were well matched with the catalytic products of the FhTPS proteins encoded by the highly expressed *FhTPS* genes, it is reasonable to deduce that their specific expression profiles might account for levels of metabolite biosynthesis and emission. In order to verify this hypothesis, the patterns of emission of the major volatile terpenes and the expression of *FhTPS* genes were compared to determine any positive relationships. The release of linalool in flowers of both *Freesia* cultivars was synchronized with the expression of *FhTPS1* in three flower tissues (petal, pistil, and stamen), whereas the expression of *FhTPS4* was only consistent with the release of linalool in calyx and torus. Expression of *FhTPS2* showed a synchronous relationship with the emission of α-terpineol in Red River^®^. *FhTPS6* was also expressed in the two cultivars at relatively high levels. In Red River^®^, its expression coincided with the release of *cis*-ocimene and *trans*-ocimene, and might also be mainly responsible for the emission of selinene from Ambiance, according to its consistent expression. *FhTPS7* was the *TPS* gene with the highest expression in Ambiance, and its expression was obviously consistent with the emissions of sesquiterpene, especially copaene. *FhTPS8* was expressed in Ambiance at a lower level, and its expression was found to be correlated with the release of another major sesquiterpene, α-gurjunene (Fig. 8). Finally, in order to confirm whether the correlation between the expression of the *FhTPS* genes and the release of the major volatile terpenes was significant, Pearson correlation evaluation was performed, and significant values (*P*<0.01 or *P*<0.05) were observed (Supplementary Tables S6 and S7).

## Discussion

### Genes in clades TPS-a, TPS-b, and TPS-g have diversified and diverged functionally, probably from a common angiosperm-specific ancestor

In the past two decades, terpene synthases have been extensively examined in terrestrial plants, and are usually divided into seven clades, designated as TPS-a, TPS-b, TPS-c, TPS-d, TPS-e/f, TPS-g, and TPS-h. TPS-a, TPS-b, and TPS-g are recognized as angiosperm-specific clades (Chen *et al.*, 2011). In this study, all the TPS proteins responsible for the biosynthesis of volatile terpenes in the cultivars studied were clustered into the angiosperm-specific TPS clades. FhTPS1, FhTPS2, FhTPS3, and FhTPS5 were grouped in the TPS-b clade, containing the RRX_8_W motif in the N-terminal region for monoterpene cyclization, which is commonly found in angiosperm-specific monoterpene synthases (Hyatt *et al.*, 2007; Chen *et al.*, 2011). Correspondingly, biochemical analysis showed that FhTPS1 and FhTPS2 had the capacity to convert GPP to considerable amounts of monoterpene, mainly catalyzing the formation of linalool and α-terpineol, respectively. In contrast, neither monoterpene nor sesquiterpene products were detected in the *in vitro* reaction system supplemented with FhTPS3 and FhTPS5 using either GPP or FPP as substrate, or in transgenic tobacco leaves. The loss of catalytic abilities of FhTPS3 and FhTPS5 might be a consequence of subfunctionalization or neofunctionalization after duplication (Rensing, 2014).

In addition, FhTPS6, FhTPS7, and FhTPS8 were found to be grouped into the TPS-a clade, which is composed of angiosperm-specific sesquiterpene synthases. As expected, these three TPS proteins were found to be capable of generating sesquiterpenes using FPP as substrate. Regarding the TPS-g clade, previous studies have shown that a prominent feature is the prevalence of acyclic products, because of the lack of an RRX_8_W motif in this clade (Dudareva *et al.*, 2003). TPS proteins of the TPS-g subfamily identified from grapevine were shown to produce acyclic monoterpenes, sesquiterpenes, and diterpenes specifically (Martin *et al.*, 2010). In our study, FhTPS4 was classified into the TPS-g clade. As with other members of this clade, FhTPS4 lacks the structural feature of the RRX_8_W motif at its N-terminus and could synthesize two kinds of acyclic monoterpene and sesquiterpene, linalool and nerolidol, respectively.

As *Freesia* TPS proteins from clades TPS-a, TPS-b, and TPS-g share the common substrate GPP, and TPS proteins from clades TPS-a and TPS-g were able to catalyze the formation of both monoterpenes and sesquiterpenes simultaneously using two kinds of substrate, it is reasonable to deduce that these angiosperm-specific *FhTPS* genes might have evolved from a common ancestor. Previous studies have shown that monoterpene and sesquiterpene synthases of angiosperms probably evolved from ancestral TPS proteins in the TPS-d subfamily through neofunctionalization (Chen *et al.*, 2011; Matsuba *et al.*, 2013). Different mono-, sesqui-, and di-*TPS* genes encoding enzymes for the synthesis of conifer-specialized terpenes have been found to belong to the gymnosperm-specific TPS-d subfamily (Martin *et al.*, 2004). Thus, it is plausible that the angiosperm-specific TPS-a, TPS-b, and TPS-g clades are substantially functionally divergent from the TPS-d clade and have diversified evolutionarily after the split between gymnosperms and angiosperms.

### Flowers of *Freesia* synthesize an abundance of volatile terpenes in reactions catalyzed by TPS proteins

In this study, a wide range of terpenes, including 14 monoterpenes, 14 sesquiterpenes, and 3 carotenoid derivatives, were detected in flowers at anthesis of Red River^®^ and Ambiance. Linalool was the predominant compound in both *Freesia* cultivars, accounting for 52.5% and 93.2%, respectively (Supplementary Table S3); it was mainly released from petals, pistils, and stamens, as well as smaller amounts from the calyx and torus (Supplementary Table S4). Linalool has been found to be widely synthesized in the *Freesia* genus, indicating that it has a pivotal role in *Freesia* plants (Manning and Goldblatt, 2010). It has also been identified as the major volatile terpene in flowers of many other plants using scent to attract pollinators, and it contributes to the sweet fragrance noted by humans (Yang *et al.*, 2013; Zeng *et al.*, 2015). Previous studies have demonstrated that floral scent profiles were species specific and could vary between closely related species or even between different varieties of the same species (Klahre *et al.*, 2011). Unlike the situation with the generally present linalool, other floral volatile components were found to be differentially released in the two *Freesia* cultivars; for instance, α-terpineol was another major monoterpene in Red River^®^, whereas a series of sesquiterpenes, with copaene and α-gurjunene as major components, were emitted from Ambiance. In the wild species of *Freesia*, further volatile compounds have been detected, which were not found in Red River^®^ and Ambiance. For instance, amounts of neral, geranial, citronellol, nerol, and geraniol were detected in *F. viridis*, *F. caryophyllacea*, *F. refracta*, and *F. occidentalis*. Methyl benzoate, originating from the phenylpropanoid/benzenoid biosynthetic pathway, was found only in *F. speciosa*. As for the components in common between the cultivars and wild species, different relative compositions were also observed; for example, ocimene, which was the dominant constituent (accounting for 54%) in *F. fucata*, was emitted in only small amounts from flowers of Red River^®^ (Manning and Goldblatt, 2010). Therefore, it can be concluded that species of the genus *Freesia* have the capability to synthesize a wide variety of distinct floral scents, mainly from the terpenoid biosynthetic pathway. Furthermore, as two unscented *Freesia* species, *F. laxa* and *F. grandiflora*, have different putative pollinators as well as distinct geographic distributions and natural habitats from scented species, it is reasonable to postulate that volatile components might play crucial roles in speciation and pollinator specificity in the genus *Freesia* (Manning and Goldblatt, 2010).

The multifunctionality of flower volatile components has been comprehensively reviewed previously (Schiestl, 2010, 2015; Muhlemann *et al.*, 2014; Abbas *et al.*, 2017). TPSs are responsible for generating the immense diversity of terpenes produced by plants (McGarvey and Croteau, 1995). Many TPSs have been found to be capable of producing multiple terpenes from a single prenyl diphosphate substrate *in vitro* (Martin *et al.*, 2010; Green *et al.*, 2012; Nieuwenhuizen *et al.*, 2013) or *in vivo* (Davidovich-Rikanati *et al.*, 2008; Green *et al.*, 2012), and the profile of terpenes produced by a given species usually comprises one or two compounds that dominate as major products with others as minor components. For example, complex blends of terpenes have been found in *A. thaliana* and *Medicago truncatula*, often produced only by a limited number of multiproduct TPS enzymes (Tholl *et al.*, 2005; Garms *et al.*, 2010). In the present study, five FhTPS proteins, FhTPS2, FhTPS4, FhTPS6, FhTPS7, and FhTPS8, were verified to have the ability to catalyze the formation of multiple volatile terpenes, whereas FhTPS1 was shown to be a single-product enzyme that could covert GPP to linalool, the predominant component of the floral scents in both *Freesia* cultivars studied (Table 1). A TPS enzyme that produces the same single product has also been found in *Vitis vinifera*, *Malus domestica*, and *Alstroemeria* (Pechous and Whitaker, 2004; Martin *et al.*, 2010; Aros *et al.*, 2012; Nieuwenhuizen *et al.*, 2013). In addition, FhTPS4, FhTPS6, and FhTPS7 were identified as bifunctional enzymes that could use both GPP and FPP as substrates simultaneously. To date, many *TPS* genes encoding bifunctional enzymes with a range of available substrates have been isolated and characterized (Bleeker *et al.*, 2011; Huang *et al.*, 2012; Dudareva *et al.*, 2013). For example, all tested cucumber (*Cucumis sativus*) TPS proteins were confirmed to be bifunctional enzymes that could catalyze the formation of monoterpenes and sesquiterpenes from GPP and FPP, respectively, with similar efficiency (Wei *et al.*, 2016).

### The spatial, temporal, and cultivar-specific release of volatile terpenes is controlled by the differential expression of *FhTPS* genes in *Freesia* x *hybrida*

**Fig. 9.**
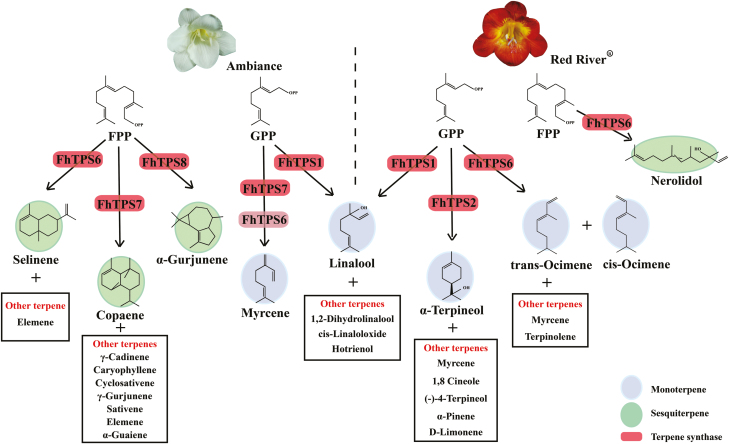
Proposed model for terpene biosynthesis in flowers of Red River^®^ and Ambiance. Briefly, FhTPS1 primarily synthesizes linalool emitted from petals, pistils, and stamens, and a small proportion of the linalool is further converted into several derivatives, such as cis-linaloloxide and 1, 2-dihydrolinalool, in both Red River^®^ and Ambiance. In the flower of Red River^**®**^, FhTPS2 catalyzes the formation of α-terpineol as well as 1,8 cineole, D-limonene, (–)-4-terpineol, and α-pinene, and FhTPS6 is associated with the formation of other monoterpenes (i.e. myrcene, limonene, *cis*-ocimene, *trans*-ocimene, and terpinolene), as well as one sesquiterpene, nerolidol. In the flower of Ambiance, nearly all of the floral sesquiterpenes are generated under the catalyzing of FhTPS7 except α-gurjunene and selinene, which are mainly synthesized by FhTPS8 and FhTPS6, respectively. Both FhTPS7 and FhTPS6 could catalyze the formation of myrcene, while *FhTPS7* might play a more important role because of its higher expression levels. FhTPS proteins are indicated by rounded rectangles. Monoterpenes and sesquiterpenes are highlighted with different backgrounds.

As shown in Fig. 3, almost all the candidate genes in the MEP and MVA pathways were expressed consistently between the two *Freesia* cultivars, indicating that they are not the determinant factors causing the differential emission profiles of the two cultivars. In contrast, the expression of *FhTPS* genes was cultivar-specific and both spatially and temporally consistent with the major terpene emission patterns (Figs 1, 2, and 6), implying that they have determining roles. Moreover, their subcellular localization and available substrate pools are essential in determining the biological significance of TPS activities *in vivo* (Chen *et al.*, 2011; Falara *et al.*, 2011). Integrating all of the information outlined above, three diagrammatic models are tentatively proposed to decipher the molecular basis of the spatial and temporal patterns of volatile terpene emission from the flowers of the two *Freesia* cultivars (Fig. 9).

In the flowers of Red River^®^ (Fig. 9), three FhTPS enzymes were responsible for the formation of the predominant flower-specific monoterpenes (Supplementary Table S8). FhTPS1 primarily synthesized the linalool that was emitted from petals, pistils, and stamens, and a small proportion of the linalool was further converted into several derivatives, such as cis-linaloloxide and 1, 2-dihydrolinalool, through oxidation and hydrolization modifications, which are commonly observed in plants (Chen *et al.*, 2011). FhTPS2 catalyzed the formation of α-terpineol as well as 1,8 cineole, D-limonene, (–)-4-terpineol, and α-pinene. Although FhTPS6 and FhTPS7 had identical catalytic products, *FhTPS6* was the functional gene *in vivo* because of its relatively higher expression levels, whereas the expression of *FhTPS7* was too low to be detected. Furthermore, FhTPS6 was deemed to be associated with the formation of other monoterpenes (myrcene, limonene, *cis*-ocimene, *trans*-ocimene, and terpinolene) as well as one sesquiterpene (nerolidol). FhTPS6 was found to be localized in the cytosol, suggesting that GPP might be available in this compartment. Previous studies have shown that longer prenyl diphosphates such as GPP and FPP could be moved from plastids to the cytosol in tomato, the grape berry exocarp, and glandular trichomes of *Stevia rebaudiana* (Gutensohn *et al.*, 2013; May *et al.*, 2013; Wölwer-Rieck *et al.*, 2014).

In the flowers of Ambiance (Fig. 9), FhTPS1 was also primarily responsible for the biosynthesis of linalool and its derivatives. In addition, *FhTPS7* was the most highly expressed sesquiterpene synthase gene, followed by *FhTPS6* and *FhTPS8*. Nearly all of the floral sesquiterpenes were generated under the catalyzing activity of the bifunctional mono-/sesquiterpene synthase FhTPS7 except α-gurjunene and selinene, which were mainly synthesized by FhTPS8 and FhTPS6, respectively (Supplementary Table S8). Despite the apparent *in vitro* enzymatic activities catalyzing the formation of significant amounts of monoterpenes, only myrcene was found among the volatile compounds. This phenomenon can be explained by the substrate selectivity of FhTPS7: FPP was shown to be more efficiently recognized by FhTPS7 than GPP (Table 2). On the other hand, FhTPS7 lacked the transit peptide at the N-terminus, meaning that the protein was retained in the cytosol, which facilitated it converting the available FPP to sesquiterpenes. A similar finding was reported in the snapdragon (*Antirrhinum majus*): AmNES/LIS-2 is plastid localized and responsible for the biosynthesis of linalool, whereas the transit peptide-lacking AmNES/LIS-1 is involved in the formation of nerolidol in the cytosol (Nagegowda *et al.*, 2008). Finally, it should be noted that FhTPS1 is responsible for the formation of linalool in Ambiance, as in Red River^®^.

As well as being detected in petal, pistil, and stamen, linalool was detected in torus and calyx, in an emission pattern synchronous with the expression of *FhTPS4*. Biochemical analysis also supported the possibility that FhTPS4 is specifically associated with linalool biosynthesis in these two floral tissues (Supplementary Fig. S18 and Table S8). Consistent with other TPS-g-clade proteins from angiosperms, FhTPS4 is also capable of catalyzing the formation of nerolidol in the presence of FPP (Yuan *et al.*, 2008; Chen *et al.*, 2011; Green *et al.*, 2012). However, nerolidol was not detected in torus and calyx in our study, which could be ascribed to the plastid subcellular localization of FhTPS4 because less GPP has been reported to be available in this organelle (Kitaoka *et al.*, 2015).

In conclusion, this study provides a molecular basis for the production of volatile terpenes in *Freesia* flowers. The characterization of key *TPS* genes responsible for the formation of major terpenes is potentially a step towards improving fragrance—and hence the ornamental and economic value—of other, unscented, horticultural plants. Furthermore, we hope it will also pave the way to understanding the evolutionary and environmental significance of the volatile terpenes in *Freesia* species and other petaloid monocots.

## Supplementary data

Supplementary data are available at *JXB* online.

Table S1. Primers used in the study.

Table S2. TPS proteins from other plant species used in phylogenetic analysis.

Table S3. Composition and contents of volatile compounds released from flowers in different developmental stages.

Table S4. Composition and contents of volatile compounds released from different floral tissues.

Table S5. Information on *FhTPS* genes isolated from flowers of *Freesia* x *hybrida* cultivars.

Table S6. Correlation analysis between gene expression and volatiles in different flower tissues of *Freesia* cultivars Red River^®^ and Ambiance

Table S7. Correlation analysis between gene expression and volatiles for fully opened flowers of *Freesia* cultivars Red River^®^ and Ambiance.

Table S8. Summary of FhTPSs in the proposed model to explain volatile terpene biosynthesis in flowers of *Freesia* cultivars Ambiance and Red River^®^.

Fig. S1. Flower developmental stages and different tissues of *Freesia* cultivar Red River^®^.

Fig. S2. Flower developmental stages and different tissues of *Freesia* cultivar Ambiance.

Fig. S3. Alignment of deduced amino acid sequences of FhTPS1 in cultivars Red River^®^ and Ambiance.

Fig S4. Alignment of deduced amino acid sequences of FhTPS2 in cultivars Red River^®^ and Ambiance.

Fig. S5. Alignment of deduced amino acid sequences of FhTPS3 in cultivars Red River^®^ and Ambiance.

Fig. S6. Alignment of deduced amino acid sequences of FhTPS4 in cultivars Red River^®^ and Ambiance.

Fig. S7. Alignment of deduced amino acid sequences of FhTPS6 in cultivars Red River^®^ and Ambiance

Fig. S8. Alignment of deduced amino acid sequences of FhTPS8 in cultivars Red River^®^ and Ambiance.

Fig. S9. Genomic structures of the *FhTPS* genes in cultivars Red River^®^ and Ambiance.

Fig. S10. Conserved residue analysis and subcellular localization of FhTPS proteins in two cultivars of *Freesia* x *hybrida*.

Fig. S11. Three-dimensional model of the structure of FhTPSs.

Fig. S12. Expression patterns of *FhTPS* genes at five developmental stages in *Freesia* cultivars Red River^®^ and Ambiance.

Fig. S13. Expression patterns of *FhTPS* genes in five flower tissues of cultivars Red River^®^ and Ambiance.

Fig. S14. Stability analysis of 18S rRNA in two cultivars of *Freesia* x *hybrida*.

Fig. S15. Detection of FhTPS proteins in *E. coli* strain BL21 (DE3).

Fig. S16. *In vitro* enzymatic activity analysis of FhTPS proteins using GPP as substrate.

Fig. S17. *In vitro* enzymatic activity analysis of FhTPS proteins using FPP as substrate.

Fig. S18. Proposed model of terpene biosynthesis in torus and calyx in both *Freesia* cultivars.

Fig. S19. *In vitro* assay of authentic standards by GC-MS.

Supplementary Tables and Figures
