# Supplementary material for: Identification and characterization of terpene synthase genes accounting for volatile terpene emissions in flowers of Freesia x hybrida
Source: J Exp Bot. 2018 Jun 12;69(18):4249–65. doi: 10.1093/jxb/ery224 (PMC6093421; doi:10.1093/jxb/ery224)
Supplement: Supplementary Tables and Figures [file ery224_suppl_supplementary_tables_and_figures.pdf]

Table S1. Primers used in the study

		Forward(5'-3')	Reverse(5'-3')
cDNAs/<br>DNAs	*FhTPS1*	ATGGCTCTCTTGCCGTGTCTTC	TTAGAGGGGAATGGGTTCAA
	FhTPS2	ATGGCGTGTCTTCCATTCCA	TTAGAGGGAAACAGGTTGTA
	FhTPS3	ATGGGGACAGAAATGGCACTC	CTATTTCATGGGCTTAAAA
	FhTPS4	ATGACTACCTTCTCAAAGAT	TTATATGCTCTTGAAAAGCA
	FhTPS5	ATGGCTTTCTTGCCGTGCCT	TTAGAGGGGAATAGGTTCAAT
	FhTPS6	ATGGAGTCAGTGCTGCTGGG	CTAGAAATAGTCATCTTCGA
	FhTPS7	ATGGAGTCAGTACTGCTGAG	CTAGTAGTAGTCCCCCGGGA
	FhTPS8	ATGGAGGTTGTGAGTGCTGGA	TCAATTCTGAATTGGCCAAAC
Generation of constructs used in subcellular localization	*FhTPS1*	GATTACGCTCATATGATGGCTCTCT<br>TGCCGTGTCT	GCTCACCATGAGCTCGAGGGGAAT<br>GGGTTCAA
	FhTPS2	GATTACGCTCATATG<br>ATGGCGTGTCTTCCATTCCA	GCTCACCATGAGCTC<br>TTAGAGGGAAACAGGTTGTA
	FhTPS3	GATTACGCTCATATGATGGGGACA<br>GAAATGGCAC	GCTCACCATGAGCTCTTTCATGGG<br>CTTAAAAA
	FhTPS4	GATTACGCTCATATG<br>ATGACTACCTTCTCAAAGAT	GCTCACCATGAGCTC<br>TTATATGCTCTTGAAAAGCA
	FhTPS5	GATTACGCTCATATGGCTTTCTTGC<br>CGTGCCTTTC	GCTCACCATGAGCTCGTTGACTTG<br>CTCATTCCCCA
	FhTPS6	GATTACGCTCATATG<br>ATGGAGTCAGTGCTGCTGGG	GCTCACCATGAGCTC<br>CTAGAAATAGTCATCTTCGA
	FhTPS7	GATTACGCTCATATGATGGAGTCAG<br>TACTGCTGAG	GCTCACCATGAGCTCGTAGTAGTC<br>CCCCGGGA
	FhTPS8	GATTACGCTCATATGATGGAGGTTG<br>TGAGTGCTGG	GCTCACCATGAGCTCATTCTGAATT<br>GGCCAAA
Generation of constructs used in Heterologous expression in *Escherichia coli*	*FhTPS1*	CAAGGATCC<br>ATGGCTCTCTTGCCGTGTCTTC	CAAGAGCTC<br>TTAGAGGGGAATGGGTTCAA
	FhTPS2	CAAGGATCC<br>ATGGCGTGTCTTCCATTCCA	CAAGAGCTC<br>TTAGAGGGAAACAGGTTGTA
	FhTPS3	CAAGGATCC<br>ATGGGGACAGAAATGGCACTC	CAAGAGCTC<br>CTATTTCATGGGCTTAAAA
	FhTPS4	CAAGGATCC<br>ATGACTACCTTCTCAAAGAT	CAAGAGCTC<br>TTATATGCTCTTGAAAAGCA
	FhTPS5	CAAGGATCC<br>ATGGCTTTCTTGCCGTGCCT	CAAGAGCTC<br>TTAGAGGGGAATAGGTTCAAT
	FhTPS6	CAAGGATCC<br>ATGGAGTCAGTGCTGCTGGG	CAAGAGCTC<br>CTAGAAATAGTCATCTTCGA
	FhTPS7	CAAGGATCC<br>ATGGAGTCAGTACTGCTGAG	CAAGAGCTC<br>CTAGTAGTAGTCCCCCGGGA

	FhTPS8	CAAGGATCC ATGGAGGTTGTGAGTGCTGGA	CAAGAGCTC TCAATTCTGAATTGGCCAAAC
qRT-PCR	*FhTPS1*	CACTACAAACCTTACCGCCAACA	GCTTTACAAAGATCTGCCCACGA
	FhTPS2	CGTCTCTGCCTTCTTCTCACT	ATGGTTTCCTCATCTTCCTGC
	FhTPS3	CTTCTTTCAACACTCCCTAC	TAACACCACTGCTCCTAACT
	FhTPS4	GAGGTTTCTCTTTCTTTTCG	GAGTCCAGTTTTCCTTTGTC
	FhTPS5	CTGTCTCTTCGCTCTTCCCG	GTTGACTTGCTCATTCCCCA
	FhTPS6	TGGAGGGAGGAAGGGTATGT	GCACGATTTTTGGAATGTTG
	FhTPS7	ACGACATCTTCTCTGCCG	CACAACCTTCCATCCATC
	FhTPS8	TCCGTTTCCGATTGTTGAGAC	ATTGAAGTAAGCCGATGATGT
	18s	TCCTGATACGGGGAGGTAGTGACA	ACTTGCCCTCCAATGGATCCTCG
Generation of constructs used in Heterologous expression in tobacco	*FhTPS1*	CGGGATCC ATGGCTCTCTTGCCGTGTCTTC	CGAGCTC TTAGAGGGGAATGGGTTCAA
	FhTPS2	CGGGATCC ATGGCGTGTCTTCCATTCCA	CGAGCTC TTAGAGGGAAACAGGTTGTA
	FhTPS6	CGGGATCC ATGGAGTCAGTACTGCTGAG	CGAGCTC CTAGTAGTAGTCCCCCGGGA
	FhTPS7	CGGGATCC ATGGAGTCAGTACTGCTGAG	CGAGCTC CTAGTAGTAGTCCCCCGGGA

Table S2. TPS proteins from other plant species used in phylogenetic analysis

Protein	Protein ID in NCBI	Note
AtTPS2	NP 193406.3	(E)-bate-ocimene/myrcene synthase
AtTPS14	NP 001185286.1	(±)-3S-linalool synthase
AtTPS24	NP 189209.2	1,8-cineole synthase
SlTPS5	NP 001233805.1	linalool synthase
SlTPS24	NP 111307929.1	ent-kaurene synthase
SlTPS40	NP 001234008.2	copalyl-diphosphate synthase
SlTPS8	XP 004231365.1	1,8-cineole synthase
AgPIN1	O24475.1	pinene synthase
MtTPS15	XP 003621227.1	ocimene synthase
MtTPS23	XP 003619707.1	nerolidol synthase
ZmTPS6	NP 001105674.1	(S)-beta-macrocarpene synthase
ZmTPS10	NP 001105850.1	(E)-beta farnesene synthase
ZmTPS23	ABY79213.1	(E)-beta caryophyllene synthase
VGwGerA	ADR66821.1	germacrene A synthase
VvGwBer	ADR74195.2	(E)-alpha-bergamotene synthase
VvGwgCad	ADR74199.1	Gamma-cadinene synthase
HcTPS8	AGY49283.1	linalool synthase
HcTPS7	AHJ57305.1	sabinene synthase
LaCARS	AGL98419.1	caryophyllene synthase
LaGERDS	AGL98420.1	germacrene D synthase
LaLINS	Q2XSC5.1	linalool synthase

Table S3. Composition and contents of volatile compounds released from flowers in different developmental stages

	Red River®					Ambiance				
**Compounds**	R1	R2	R3	R4	R5	A1	A2	A3	A4	A5
**Monoterpenes**										
α-Pinene	n.d	n.d	n.d	24.15±3.86	33.57±4.16	n.d	n.d	n.d	n.d	n.d
β-Pinene	n.d	n.d	n.d	42.07±5.09	25.71±2.99	n.d	n.d	n.d	n.d	n.d
Myrcene	n.d	n.d	n.d	57.33±2.61	69.21±4.26	n.d	n.d	n.d	14.83±3.42	28.54±2.52
1,8 cineole	n.d	n.d	11.38±0.92	76.54±12.15	334.28±23.30	n.d	n.d	n.d	n.d	n.d
D-Limonene	n.d	n.d	17.09±1.38	45.67±7.95	143.57±16.79	n.d	n.d	n.d	n.d	n.d
cis-Ocimene	n.d	n.d	26.17±3.68	200.67±28.58	736.42±59.13	n.d	n.d	n.d	n.d	n.d
trans-Ocimene	n.d	n.d	50.64±6.74	125.62±10.28	617.86±62.09	n.d	n.d	n.d	n.d	n.d
cis-Linaloloxide	n.d	n.d	n.d	79.67±13.14	152.14±23.59	n.d	n.d	n.d	30.67±5.19	33.63±4.61
Terpinolene	n.d	n.d	n.d	8.72±3.29	41.43±7.03	n.d	n.d	n.d	n.d	n.d
Linalool	n.d	31.25±5.38	176.32±32.12	7134.39±1096.23	18345.13±2247.65	80.50±5.33	163.41±9.01	325.76±45.98	14971.67±1375.14	24341.88±1527.14
1,2-Dihydrolinalool	n.d	n.d	n.d	n.d	21.43±4.67	n.d	n.d	n.d	121.87±12.65	162.51±20.94
(-)-4-Terpineol	n.d	n.d	n.d	22.34±4.26	35.58±2.14	n.d	n.d	n.d	n.d	n.d
Hotrienol	n.d	n.d	n.d	n.d	n.d	n.d	n.d	n.d	98.27±7.83	170.25±8.75

α-Terpineol	10.13±1.84	26.31±5.38	164.21±11.12	6981.34±891.36	8876.42±291.71	n.d	n.d	n.d	n.d	n.d
**Sesquiterpenes**										
Cyclosativene	n.d	n.d	n.d	13.29±6.22	35.83±13.85	n.d	n.d	n.d	n.d	5.38±2.15
Copaene	n.d	n.d	n.d	n.d	n.d	9.76±0.25	60.58±7.36	116.08±10.38	404.15±30.27	542.53±36.57
Elemene	n.d	n.d	n.d	n.d	n.d	1.83±0.09	10.03±1.21	15.02±1.09	40.17±2.18	58.09±8.08
α-Gurjunene	n.d	n.d	n.d	n.d	n.d	1.83±0.83	13.08±2.19	26.50±2.96	101.28±9.54	127.63±17.07
Caryophyllene	n.d	n.d	n.d	n.d	n.d	n.d	n.d	n.d	2.68±0.74	4.25±2.00
α-Guaiene	n.d	n.d	n.d	n.d	n.d	n.d	n.d	6.58±0.99	13.17±2.58	20.13±3.81
α-Patchoulene	n.d	n.d	n.d	n.d	n.d	n.d	n.d	n.d	6.83±1.01	15.75±4.50
Sativene	n.d	n.d	n.d	n.d	n.d	n.d	6.77±2.08	11.08±0.97	35.34±5.21	43.25±4.08
γ-Cadinene	n.d	n.d	n.d	n.d	n.d	n.d	n.d	n.d	21.54±2.59	93.88±11.73
γ-Gurjunene	n.d	n.d	n.d	23.47±7.32	75.57±14.67	n.d	n.d	14.78±3.28	28.67±5.28	26.63±6.96
Selinene	n.d	n.d	n.d	n.d	n.d	n.d	n.d	n.d	7.17±2.88	8.38±3.04
α-Bulnesene	n.d	n.d	n.d	n.d	n.d	n.d	11.42±3.63	20.09±2.85	65.43±9.82	96.10±9.29
Vatirenene	n.d	n.d	n.d	n.d	n.d	n.d	n.d	n.d	n.d	6.25±1.71
Nerolidol	n.d	n.d	n.d	19.33±8.23	36.21±9.35	n.d	n.d	n.d	n.d	n.d

**Carotenoid derivatives**										
Dihydro-ionone	n.d	n.d	5.27±0.43	601.26±35.97	913.02±21.23	n.d	11.09±4.21	7.27±2.94	19.67±4.51	16.50±3.47
β-Ionone	n.d	n.d	11.61±4.76	2622.19±89.02	4350.29±102.53	n.d	14.12±1.26	10.37±1.38	12.84±1.72	18.75±1.53
α-Cyclocitral	n.d	n.d	n.d	n.d	74.28±10.05	n.d	n.d	n.d	n.d	n.d

R1-R5 and A1-A5 were the flower developmental stages defined as previously studies (Li *et al*., 2016; Sun *et al*., 2016; Sun *et al*., 2016)

n.d represented tentatively undetected

Data were the means ± SD of three independent experiments, and the unit was ng g^{-1} FW (fresh weight).

Table S4. Composition and contents of volatile compounds released from different flower tissues

	Red River®					Ambiance				
**Compounds**	Petal	Pistill	Stamen	calyx	torus	Petal	Pistill	Stamen	calyx	torus
**Monoterpenes**										
α-Pinene	698.27±12.83	2768.64±173.63	255.64±37.28	n.d	n.d	n.d	n.d	n.d	n.d	n.d
β-Pinene	245.14±15.73	838.36±47.83	51.39±12.13	n.d	n.d	n.d	n.d	n.d	n.d	n.d
Myrcene	774.38±58.34	1437.19±98.42	162.59±32.57	n.d	n.d	328.78±28.12	n.d	n.d	n.d	n.d
1,8 cineole	1396.78±127.49	5268.82±289.47	885.71±57.15	n.d	n.d	n.d	n.d	n.d	n.d	n.d
D-Limonene	1801.68±78.85	5631.57±127.61	650.79±37.68	n.d	n.d	n.d	n.d	n.d	n.d	n.d
cis-Ocimene	136.48±18.87	n.d	n.d	n.d	n.d	n.d	n.d	n.d	n.d	n.d
trans-Ocimene	176.68±13.48	n.d	n.d	n.d	n.d	n.d	n.d	n.d	n.d	n.d
cis-Linaloloxide	268.63±12.57	n.d	n.d	n.d	n.d	110.47±8.37	n.d	n.d	n.d	n.d
Terpinolene	215.70±13.63	240.12±27.38	n.d	n.d	n.d	n.d	n.d	n.d	n.d	n.d

Linalool	56372.75±1389.89	69562.78±1686.61	17362.45±749.32	3906.67±197.34	2284.48±139.32	79726.54±2185.37	98650.26±2098.67	12500.67±1098.72	4238.56±209.98	3109.67±393.19
1,2-Dihydrolinalool	83.38±12.97	793.57±26.59	n.d	n.d	n.d	197.38±10.24	n.d	n.d	n.d	n.d
(-)-4-Terpineol	60.39±9.68	n.d	n.d	n.d	n.d	n.d	n.d	n.d	n.d	n.d
Hotrienol	n.d	n.d	n.d	n.d	n.d	63.96±6.91	n.d	n.d	n.d	n.d
α-Terpineol	8772.33±503.38	21687.38±729.96	8975.48±305.21	n.d	n.d	n.d	n.d	n.d	n.d	n.d
**Sesquiterpenes**										
Cyclosativene	29.68±3.89	n.d	n.d	n.d	n.d	11.78±1.98	n.d	n.d	n.d	n.d
Copaene	n.d	n.d	n.d	n.d	n.d	1135.73±106.38	2353.83±102.39	637.51±67.93	n.d	n.d
Elemene	n.d	n.d	n.d	n.d	n.d	137.67±13.94	1093.71±49.92	140.09±15.37	n.d	n.d
α-Gurjunene	n.d	n.d	n.d	n.d	n.d	286.67±16.32	1207.28±59.01	220.72±23.01	n.d	n.d
Caryophyllene	n.d	n.d	n.d	n.d	n.d	13.73±2.98	n.d	n.d	n.d	n.d
α-Guaiene	n.d	n.d	n.d	n.d	n.d	23.45±3.29	n.d	n.d	n.d	n.d
α-Patchoulene	n.d	n.d	n.d	n.d	n.d	12.09±2.63	n.d	n.d	n.d	n.d

Sativene	n.d	n.d	n.d	n.d	n.d	80.93±10.37	596.28±9.12	186.41±15.23	n.d	n.d
γ-Cadinene	n.d	n.d	n.d	n.d	n.d	45.02±6.29	n.d	210.14±10.29	n.d	n.d
γ-Gurjunene	74.97±3.09	n.d	n.d	n.d	n.d	67.37±4.29	n.d	n.d	n.d	n.d
Selinene	n.d	n.d	n.d	n.d	n.d	21.38±1.31	n.d	n.d	n.d	n.d
α-Bulnesene	n.d	n.d	n.d	n.d	n.d	119.38±10.23	665.12±30.12	322.45±20.91	n.d	n.d
Vatirenene	n.d	n.d	n.d	n.d	n.d	25.03±3.01	n.d	n.d	n.d	n.d
Nerolidol	60.67±4.78	n.d	n.d	n.d	n.d	n.d	n.d	n.d	n.d	n.d
**Carotenoid derivatives**										
Dihydro-Ionone	1002.67±34.78	1078.67±46.89	1524.96±39.78	n.d	n.d	n.d	n.d	n.d	n.d	n.d
β-Ionone	4667.69±397.97	2263.18±148.98	4274.88±127.97	n.d	n.d	23.17±2.39	n.d	n.d	n.d	n.d
α-Cyclociral	335.46±30.78	n.d	n.d	n.d	n.d	n.d	n.d	n.d	n.d	n.d

n.d represented tentatively undetected

Data were the means ± SD of three independent experiments, and the unit was ng g^{-1} FW (fresh weight).

Table S5. Information of *FhTPS* genes isolated from flowers of *Freesia* cultivars

**Candidate transcripts**	***FhTPS* genes**	**Protein sequence length**	**Top *Arabidopsis* BLAST match**	**Top BLAST match excluding *Arabidopsis***	**Homology (%)**	**Function prediction**
Unigene_125076	*FhTPS1*	592 residues	NP_179998.1 terpene synthase 10 [*Arabidopsis thaliana*]	AMT81307.1 myrcene synthase [*Lilium sp.BT-2016*]	44[a],53[b]	Monoterpene synthase
Unigene_125270	*FhTPS2*	595 residues	NP_189212.1 terpene synthase-like sequence-1.8-cineole [*Arabidopsis thaliana*]	AMT81307.1 myrcene synthase [*Lilium sp.BT-2016*]	44[a],54[b]	Monoterpene synthase
Unigene_125191	*FhTPS3*	590 residues	NP_567511.3 terpene synthase 03 [*Arabidopsis thaliana*]	OAY64179.1Alpha-terpineol synthase,chloroplastic [*Ananas comosus*]	43[a],55[b]	Monoterpene synthase
Unigene_116598	*FhTPS4*	566 residues	NP_176361.2 terpene synthase14[*Arabidopsis thaliana*]	ADR74212.1(3S)-linalool/(E)-nerolidol synthase [*Vitis vinfera*]	45[a],50[b]	Monoterpene synthase/Sesquiterpene synthase
Unigene_110102	*FhTPS5*	607 residues	NP_189212.1 terpene	AMT81307.1 myrcene synthase	45[a],53[b]	Monoterpene synthase

			synthase-like sequence-1.8-cineole protein [*Arabidopsis thaliana*]	[*Lilium sp.BT-2016*]		
Unigene_122329	*FhTPS6*	566 residues	NP_197784.2 terpene synthase 21[*Arabidopsis thaliana*]	AAC315702.2 sesquiterpene synthase [*Elaeis oleifera*]	36[a],55[b]	Sesquiterpene synthase
Unigene_105518	*FhTPS7*	570 residues	NP_197784.2 terpene synthase 21[*Arabidopsis thaliana*]	AAC315702.2 sesquiterpene synthase [*Elaeis oleifera*]	35[a],51[b]	Sesquiterpene synthase
Unigene_80141	*FhTPS8*	566 residues	NP_197784.2 terpene synthase 21[*Arabidopsis thaliana*]	All32473.1 terpene synthase [*Populus trichocarpa*]	35[a],44[b]	Sesquiterpene synthase

a% Similarity to *Arabidopsis*.

b% Similarity to other plant sequence

Table S6. Correlation analysis between gene expression and volatiles in different flower tissues of Red River® and Ambiance

	FhTPS1		*FhTPS2*		*FhTPS4*		*FhTPS6*		*FhTPS7*		*FhTPS8*	
	Red River®	Ambiance	Red River®	Ambiance	Red River®	Ambiance	Red River®	Ambiance	Red River®	Ambiance	Red River®	Ambiance
α-Pinene	---	---	0.374	NA	---	---	---	---	---	---	---	---
β-Pinene	---	---	---	---	---	---	---	---	---	---	---	---
Myrcene	---	---	0.623*	-0.306	---	---	0.296	0.477	NA	0.445	---	---
1,8 cineole	---	---	0.377	NA	---	---	---	---	---	---	---	---
D-Limonene	---	---	0.438	NA	---	---	0.114	NA	NA	NA	---	---
cis-Ocimene	---	---	---	---	---	---	0.838**	NA	NA	NA	---	---
trans-Ocimene	---	---	---	---	---	---	0.837**	NA	NA	NA	---	---
cis-Linaloloxide	---	---	---	---	---	---	---	---	---	---	---	---
Terpinolene	---	---	---	---	---	---	0.479	NA	NA	NA	---	---
Linalool	0.700**	0.692**	---	---	0.763**	0.929**	0.514*	0.703**	NA	0.482	---	---
1,2-Dihydrolinalool	---	---	---	---	---	---	---	---	---	---	---	---
(-)-4-Terpineol	---	---	---	---	---	---	---	---	---	---	---	---
Hotrienol	---	---	---	---	---	---	---	---	---	---	---	---
α-Terpineol	---	---	0.553*	NA	---	---	---	---	---	---	---	---
Cyclosativene	---	---	---	---	---	---	---	---	NA	0.437	---	---
Copaene	---	---	---	---	---	---	NA	0.693**	NA	0.516*	---	---
Elemene	---	---	---	---	---	---	NA	0.423	NA	0.345	---	---
α-Gurjunene	---	---	---	---	---	---	---	---	---	---	NA	0.929**
Caryophyllene	---	---	---	---	---	---	NA	0.521*	NA	0.431	---	---
α-Guaiene	---	---	---	---	---	---	---	---	NA	0.440	---	---
α-Patchoulene	---	---	---	---	---	---	---	---	---	---	---	---
Sativene	---	---	---	---	---	---	---	---	NA	0.378	---	---

γ-Cadinene	---	---	---	---	---	---	---	---	NA	0.709**	---	---
γ-Gurjunene	---	---	---	---	---	---	---	---	---	---	---	---
Selinene	---	---	---	---	---	---	NA	0.544*	---	---	NA	-0.306
α-Bulnesene	---	---	---	---	---	---	---	---	---	---	---	---
Vatirenene	---	---	---	---	---	---	---	---	---	---	---	---
Nerolidol	---	---	---	---	---	---	0.837**	NA	NA	NA	-0.301	NA

--- represented that volatile terpenes of *Freesia hybrid* were undetected in enzymatic assay of FhTPS

NA represented no Correlation

** p<0.01   * p<0.05

Table S7. Correlation analysis between gene expression and volatiles for fully opened flowers of Red River® and Ambiance

	FhTPS1	*FhTPS2*	*FhTPS6*	*FhTPS7*	*FhTPS8*
α-Pinene	---	0.997**	---	---	---
β-Pinene	---	---	---	---	---
Myrcene	---	0.985**	0.975**	-0.962	---
1,8 cineole	---	0.998**	---	---	---
D-Limonene	---	0.998**	0.976**	-0.969	---
cis-Ocimene	---	---	0.977**	-0.974	---
trans-Ocimene	---	---	0.977**	-0.971	---
cis-Linaloloxide	---	---	---	---	---
Terpinolene	---	---	0.971**	-0.959	---
Linalool	0.832*	---	-0.819	0.576	---
1,2-Dihydrolinalool	---	---	---	---	---
(-)-4-Terpineol	---	---	---	---	---
Hotrienol	---	---	---	---	---
α-Terpineol	---	0.992**	---	---	---
Cyclosativene	---	---	---	-0.853	---
Copaene	---	---	-0.970	0.983**	---
Elemene	---	---	-0.959	0.981**	---
α-Gurjunene	---	---	---	---	0.871*
Caryophyllene	---	---	-0.848	0.902**	---
α-Guaiene	---	---	---	0.976**	---
α-Patchoulene	---	---	---	---	---
Sativene	---	---	---	0.983**	---
γ-Cadinene	---	---	---	0.982**	---
γ-Gurjunene	---	---	---	---	---

Selinene	---	---	-0.892	---	0.947**
α-Bulnesene	---	---	---	---	---
Vatirenene	---	---	---	---	---
Nerolidol	---	---	0.956**	-0.933	-0.707

--- represented that volatile terpenes of *Freesia hybrid* were undetected in enzymatic assay of FhTPS

NA represented no Correlation

** $p<0.01$ * $p<0.05$

Table S8. Summary of FhTPSs in the proposed model to explain volatile terpene biosynthesis in flowers of Ambiance and Red River®

Gene	Subcellular location		Tissue specific expression		Enzymatic products	
	Chloroplast	Cytoplasm	Red River®	Ambiance	Red River®	Ambiance
FhTPS1	Y	---	Petal/Pistil/Stamen	Petal/Pistil/Stamen	Linalool	Linalool
FhTPS2	Y	---	Petal/Pistil/Stamen	---	α-Terpineol/ Myrcene	---
FhTPS4	Y	---	Calyx/Torus	Calyx/Torus	Linalool	Linalool
FhTPS6	---	Y	Petal/Pistil/Stamen	Petal/Pistil/Stamen	cis-Ocimene/ trans-Ocimene/Myrcene / Nerolidol	Selinene / Myrcene/ Elemene
FhTPS7	---	Y	---	Petal/Pistil/Stamen	---	Copaene/γ-Cadinene/ Caryophyllene/ Cyclosativene/γ-Gurjunene/ Sativeneα-Guaiene/ Elemene/ Myrcene
FhTPS8	---	Y	---	Petal/Pistil/Stamen	---	α-Gurjunene

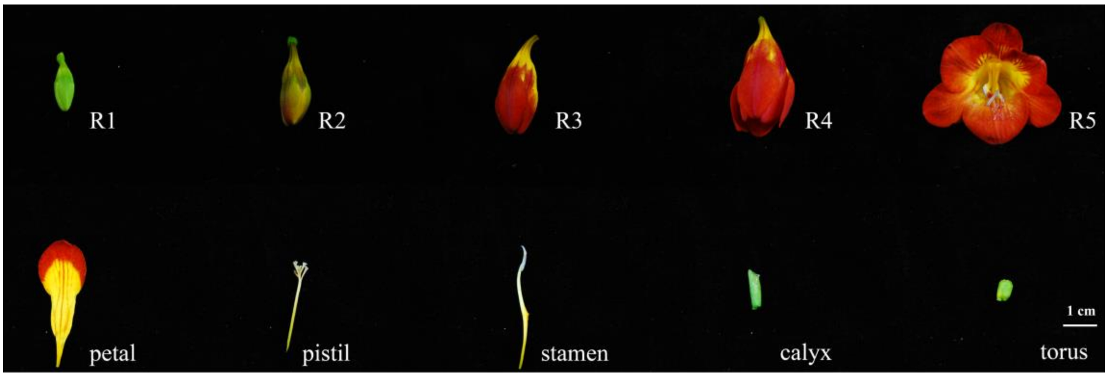

Fig. S1. Flower developmental stages and different tissues of Red River®.

The flower developmental stages and flower tissues were defined as in previous studies (Li *et al*., 2016; Sun *et al*., 2016; Sun *et al*., 2016)

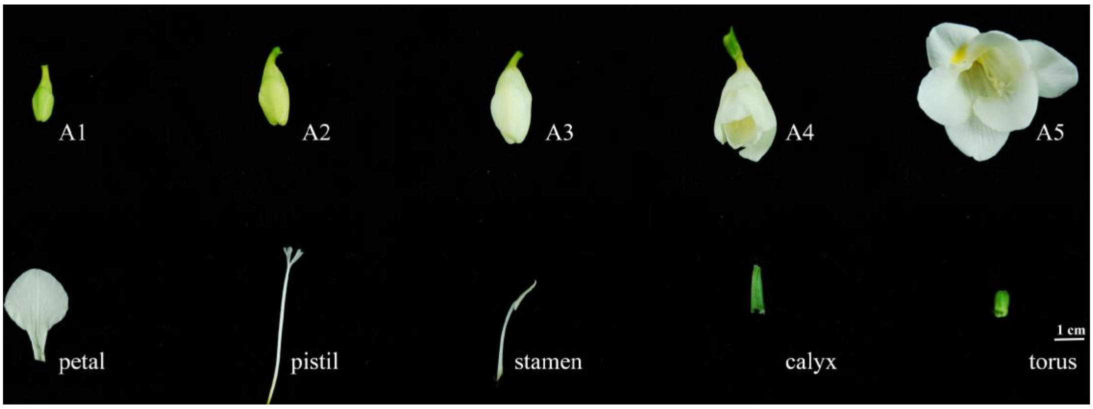

Fig. S2. Flower developmental stages and different tissues of Ambiance.

The flower developmental stages and flower tissues of Ambiance were defined according to that of Red River®, as in previous studies (Li *et al.*, 2016; Sun *et al.*, 2016; Sun *et al.*, 2016).

```
FhTPS1 (Red River®)  MALLPCLPSQFPCSPVTGFVRLPLLSSRRSSKGVQSSNYRFRCCINTGTSVSQPLRRAASYPQNIWDDSYIQALNCGYMGDEQVNEIRKLKEEVGQLFSDSKEILYQIELIDELQQLGVA
FhTPS1 (Ambiance)    MALLPCLPSQFPCSPVTGFVRLPLLSSRRSSKGVQSSNYRFRCCINTGTSVSQPLRRAASYPQNIWDDSYIQALNCGYMGDEQVNEIRKLKEEVGQLFSDSKEILYQIELIDELQQLGVA
                     ************************************************************************************************************************
FhTPS1 (Red River®)  YHFQDEIKDKLSTIFCSLEKTSLFMENDLKATSLVFRLLREHGFHASADIFNNFRENKGNFKSCLKNDMEGMINLYEASFFAVEGENQLDEARVFATEHLRHLSESLVEASLRERVAHAL
FhTPS1 (Ambiance)    YHFQDEIKDKLSTIFCSLEKTSLFMENDLKATSLVFRLLREHGFHASADIFNNFRENKGNFKSCLKNDMEGMINLYEASFFAVEGENQLDEARVFATEHLRHLSESLVEASLRERVAHAL
                     ************************************************************************************************************************
FhTPS1 (Red River®)  ELPLHFRMSRLHTRWFIDWYEKKVDKNSNLCRLAKLDFNFVQNIYKRELKELSRWWTNLGLGQKLSFARDRLVENYLFVIGWAFEPKLWQNREAMTMANCLVTTLDDIYDVYGSLDELEL
FhTPS1 (Ambiance)    ELPLHFRMSRLHTRWFIDWYEKKVDKNSNLCRLAKLDFNFVQNIYKRELKELSRWWTNLGLGQKLSFARDRLVENYLFVIGWAFEPKLWQNREAMTMANCLVTTLDDIYDVYGSLDELEL
                     ************************************************************************************************************************
FhTPS1 (Red River®)  FTDAVNRWDAEAIEQLPDYMKTCIMALFNTTNLTANKIMYSKGVNIIPQLRRSWADLCKAYLVEAKWYHSGYMPTLEEYLDTAWISISGPVVLTQAYCTSENITDEALKCYNFYPDVVRQ
FhTPS1 (Ambiance)    FTDAVNRWDAEAIEQLPDYMKTCIMALFNTTNLTANKIMYSKGVNIIPQLRRSWADLCKAYLVEAKWYHSGYMPTLEEYLDTAWISISGPVVLTQAYCTSENITDEALKCYNFYPDVVRQ
                     ************************************************************************************************************************
FhTPS1 (Red River®)  SSMISRLWNDLATSTAEMERGDVPKSIQCYMHEKGVSEEVAREHIRDMIVSISKKFDYDCISNSSIAESLKSVALDVHRMSQCVYQYEDGYGEQGHQKREQVISLLFEPIPL
FhTPS1 (Ambiance)    SSMISRLWNDLATSTAEMERGDVPKSIQCYMHEKGVSEEVAREHIRDMIVSISKKFDYDCISNSSIAESLKSVALDVHRMSQCVYQYEDGYGEQGHQKREQVISLLFEPIPL
                     ****************************************************************************************************************
```

Fig. S3. Alignment of deduced amino acid sequences of FhTPS1 in Red River® and Ambiance.

```
FhTPS2   (Red River®)   MACLPFHYTTYSRSPAGILFRSSLPSSHCRARRSRSNESANRIRCCNNTQISQPLRRTANYPPTIWENSYIQEPNTDYMQEDEETIEIGKLKEYVMTRLIISNSDQIELIDTLQQLGVAY
FhTPS2   (Ambiance)     MACLPFHYTTYSRSPAGILFRSSLPSSHCRARRSRSNESANRIRCCNNTQISQPLRRTANYPPTIWENSYIQELNTDYMQEDEETIEIGKLKEYVMTRLIISNSDQIELIDTLQQLGVAY
                        ************************************************************************* **********************************************

FhTPS2   (Red River®)   HFQEEIQNILATIFCSIKKIIPTIHNDIYATALLFRLLREKGFHVSTNIFNNFKEEGGTFKACLKNDIKGMLSLYEASFLAVEGENELDEARLFATECLKHTMENSLSLEPSMKERIVHA
FhTPS2   (Ambiance)     HFQEEIQNILATIFCSIKKIIPTIHNDIYATALLFRLLREKGFHVSTNIFNNFKEEGGTFKACLKNDIKGMLSLYEASFLAVEGENELDEARLFATECLKHTMENSLSLEPSMKERIVHA
                        ************************************************************************************************************************

FhTPS2   (Red River®)   LELPLHWRMSRLHSRWFIDQYEKDEKMNPTLLRLAKLDFNFVQTIYKRELKELSRWWSNLDLLGDKLGFARDRLVENYLWTVGSAFEPKFWQSREALTKANCLITTIDDIYDVYGTLDEL
FhTPS2   (Ambiance)     LELPLHWRMSRLHSRWFIDQYEKDEKMNPTLLRLAKLDFNFVQTIYKRELKELSRWWSNLDLLGDKLGFARDRLVENYLWTVGSAFEPKFWQSREALTKANCLITTIDDIYDVYGTLDEL
                        ************************************************************************************************************************

FhTPS2   (Red River®)   VLFTDVADRWDVNAIEQLPDYMKTCLLALFNTTNDTAYKILNLKGVIIIPQLKKVWADLCKAYLVEAKWYHSGYMPTLEEYLDNGWISISGHVALAHAFCTSEDITYRALQCYNQLSPNL
FhTPS2   (Ambiance)     VLFTDVVDRWDVNAIEQLPDYMKTCLLALFNTTNDTAYKILNLKGVIIIPQLKKVWADLCKAYLVEAKWYHSGYMPTLEEYLDNGWISISGHVALAHAFCTSEDITYRALQCYNQLSPNL
                        ******.*****************************************************************************************************************

FhTPS2   (Red River®)   LFHSSVIVRLVDDLATSTAELERGDVPKAIQCYMKQNRVSEEVAREKIKEMIVSTWEKLNGDLIATSSVVESFQSVALNFPRMAQCIYQYGDGYGDPTQKTKDQIVSLLIQPVSL
FhTPS2   (Ambiance)     VFHSSVIVRLVDDLATSTAELERGDVPKAIQCYMKQNRVSEEVAREKIKEMIVSTWEKLNGDLIATSSVVESFQSVALNFPRMAQCIYQYGDGYGDPTQKTKDQIVSLLIQPVSL
                        :******************************************************************************************************************
```

Fig. S4. Alignment of deduced amino acid sequences of FhTPS2 in Red River® and Ambiance.

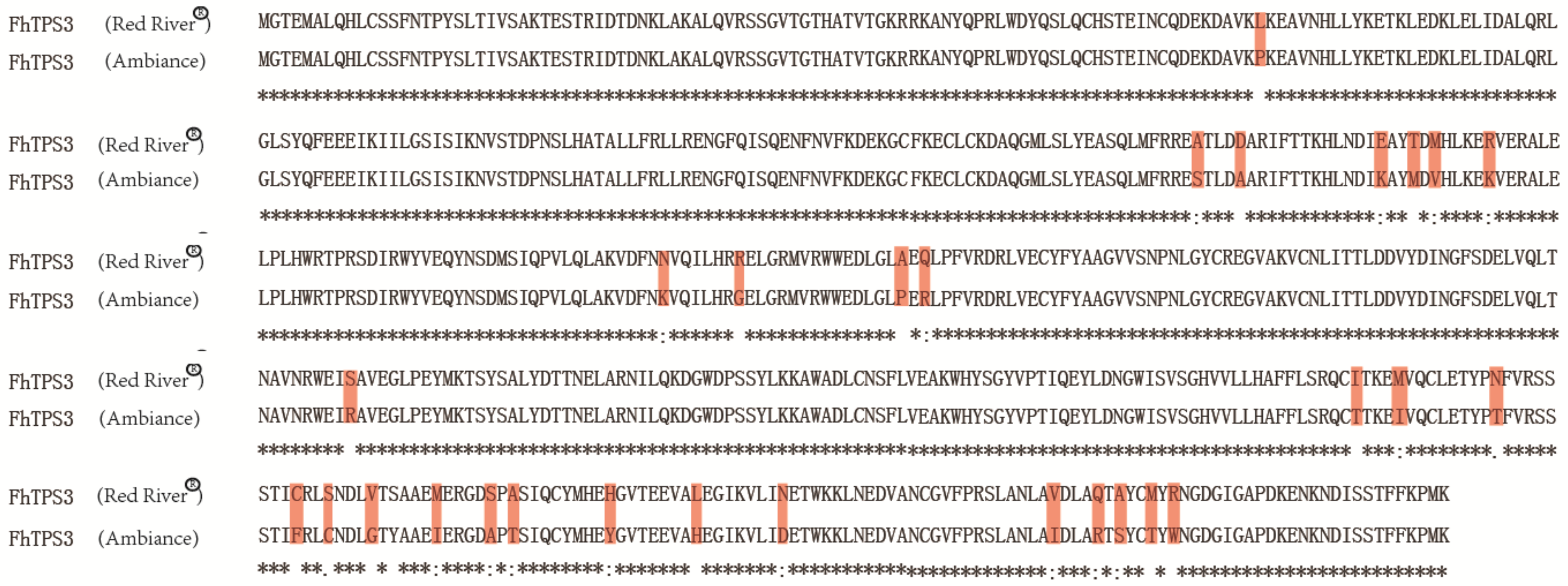

Fig. S5. Alignment of deduced amino acid sequences of FhTPS3 in Red River® and Ambiance.

```
FhTPS4 (Red River®)  MTTFSKMSASPPSPFVTTRNSGCANKTSTGTISQSNYSPQSARLVQRLGSLLTNVREDQSDMKHAENLMRVKSLFPQLEDPLECMNTIDSLQRLGIDYHFKKEIKDMLGHIYERFRQIEH
FhTPS4 (Ambiance)    MTTFSKMSASPPSPFVTTRNSGCANKTSTGTISQSHYSPQSVRLVQRLGSLLTSVREDQSDMKHAENLMRVKSLFPQLEDPLECMNTIDSLQRLGIDYHFKKEIKDMLGHIYERFRQIEH
                     ***********************************:*****.***********.******************************************************************

FhTPS4 (Red River®)  HLITGDLFEVSLSFRLLRQAGHHVSSDVFYKFIDDKGKLDSSLRTDIEGLLSLHEASYLNTGEDILYRTKEFTIEHLTSCMEHLESDGASLVEQTLKSPIHKTLSKYNSPYYINRRQEKL
FhTPS4 (Ambiance)    HLITGDLFEVSLSFRLLRQAGHHVSSDVFYKFIDDKGKLDSSLRTDIEGLLSLHEASYLNTGEDILYRTKEFTIEHLTSCMEHLESDGASLVEQTLKSPIHKTLSKYNSPYYINRRQEKL
                     ************************************************************************************************************************

FhTPS4 (Red River®)  TRYGVLNEVARVDYNQVQTIYQRELFEILSWWKEIGLVQELNFIRDQPLKWYTWSMTVLPDPQFSKCRISLTKVIAFVYIIDDIFDIYGTLEELSLFTEAIRKWELSGAETLPTYMQILY
FhTPS4 (Ambiance)    TRYGVLNEVARVDYNQVQTIYQRELFEILSWWKEIGLVQELNFIRDQPLKWYTWSMTVLPDPQFSKCRISLTKVIAFVYIIDDIFDIYGTLEELSLFTEAIRKWELSGAETLPTYMQILY
                     ************************************************************************************************************************

FhTPS4 (Red River®)  KTLYDITNEIAEATYEEHNWNPIGHLKESWARLCDAFLKEAKWFQSKKVPKADEYLANAIVSSGVYTVLLHAYFLLGEGITQENANFLKTNPTLLSSPATILRLWDDLGNAEDENQEGYD
FhTPS4 (Ambiance)    KTLYDITNEIAEATYEEHNWNPIGHLKESWARLCDAFLKEAKWFQSKKVPKADEYLANAIVSSGVYTVLLHAYFLLGEGITQENANFLKTNPTLLSSPATILRLWDDLGNAEDENQEGHD
                     **********************************************************************************************************************:*

FhTPS4 (Red River®)  GSYVEYLMQENPNYTMESSRDHVMKMISSSWEALNKECFSSSRFAPNLMAGCLNLSRMIEVMYSYDTNQNLPVLEEYITKLLFKSI
FhTPS4 (Ambiance)    GSYVEYLMQENPNYTMESSRDHVMKMISSSWEALNKECFSSSQFAPNLMAGCLNLPRMIEVMYSYDTNQNLPVLEEYITKLLFKSI
                     ******************************************:************ ******************************
```

Fig. S6. Alignment of deduced amino acid sequences of FhTPS4 in Red River® and Ambiance.

```
FhTPS6  (Red River®)  MESVLLGSPPDGTVTSSVTRRPASANYHPSVWGDYFIKQQFPPSKIQKSEAWIKQRVEELIIKIKIMLTESTDQLQEMQLIDAVQRLGVAYHFEKEIDDKLRRIHNANLDSSDLHFISLR
FhTPS6  (Ambiance)     MESVLLGSPPDGTVTSSVTRRPASANYHPSVWGDYFIKQQFPPSKIQKSEAWIKQRVEELIIKIKIMLTESTDQLQEMQLIDAVQRLGVAYHFEKEIDDKLRRIHNANLDSSDLHFISLR
                      ************************************************************************************************************************

FhTPS6  (Red River®)  FRLLRQHGYNVPSDVFNKFKDDEGNFRSSLCEQVRVLLSLYEAAYLSIPGEDILDEALEFTKRHLKYYSMESNYLEPALATHISHALQAPLRRKLERLEARQYINIYEKDDEIRNDYILE
FhTPS6  (Ambiance)     FRLLRQHGYNVPSDVFNKFKDDEGNFRSSLCEQVRVLLSLYEAAYLSIPGEDILDEALEFTKRHLKYYSMESNYLEPALATHISHALQAPLRRILERLEARQYINIYEKDDEIRNDYILE
                      ********************************************************************************************* **************************

FhTPS6  (Red River®)  FAKLDFHLLQLVHREELKSISGWWKSSGLIEKLNYARDRVAECYFWALGVYYEPCYSRARKMLTKVLLQFSLMDDTYDAYGTLEELQLYTKAIQRWNLDGVDELEECMKFQYLALYDMAK
FhTPS6  (Ambiance)     FAKLDFHLLQLVHREELKSISEWWKSSGLIEKLNYARDRVAECYFWALGVYYEPCYSRARKMLTKVLLQFSLMDDTYDAYGTLEELQLYTKAIQRWNLDGVDELEECMKFQYLALYDMAK
                      ********************* **************************************************************************************************

FhTPS6  (Red River®)  DFEDELADDNIQYRVNYLREATKNTTKAWLKEAEWREEGYVPSFEEYFTVSLPSATYPTVACVSYVGMGEIVTKEALDWIFNIPKIVQAATMITRCMDDLVSSEFERKRDHVATAIQCYM
FhTPS6  (Ambiance)     DFEDELADDNIQYRVNYLREATKNTTKAWLKEAEWREEGYVPSFEEYFTVSLPSATYPTVACVSYVGMGEIVTKEALDWIFNIPKIVQAATMITRCVDDLVSSEFERKRDHVATAIQCYM
                      ************************************************************************************************:***********************

FhTPS6  (Red River®)  KEYEGASSEDACKVIRKMVEDGWKVANQECLNQKISIHLLTKIFNLARVMETMYKEIDSYTQSTTTLKDHITLLFVEPISFEDDYF
FhTPS6  (Ambiance)     KEYEGASSEDACKVIRKMVEDGWKVANQECLNQKIPIHLLTKIFNLARVMETMYKEIDSYTQSTTTLKDHITLLFVEPISFEDDYF
                      *********************************** **************************************************
```

Fig. S7. Alignment of deduced amino acid sequences of FhTPS6 in Red River® and Ambiance.

```
FhTPS8 (Red River®)  MEVVSAGEEVVRPIAKFSPSIWGDFFINHSFPLANDKNAITLIEQRVEELKRTVKELFSTNQYITERLLLIDDLQRLGIDYRFKQEIDEALKCIHIDNDNIDDNMYLVALRFRLLRQQGY
FhTPS8 (Ambiance)    MEVVSAGEEVVRPIAKFSPSIWGDFFINHSFPLANDKNAITLIEQRVEELKRTVKELFSTNQYITERLLLIDDLQRLGIDYRFKQEIDEALKCIHIDNDNIDDNMYLVALRFRLLRQQGY
                     ************************************************************************************************************************

FhTPS8 (Red River®)  HVSSDVFSKFKDDNGSFKQEYAGNIIGLLQLYEATGVRIQEDSILDEAFDFAKYHMERSITADTVKGVLADRIRHALEMPFHRSRKRLGARYYMSIYEKDEARNDVLLELAKLDFTLLQR
FhTPS8 (Ambiance)    HVSSDVFSKFKDDNGSFKQEYAGNIIGLLQLYEATGVRIQEDSILDEAFDFAKYHMERSITADTVKGVLADRIRHALEMPFHRSRKRLGARYYMSIYEKDEARNDVLLELAKLDFTLLQR
                     ************************************************************************************************************************

FhTPS8 (Red River®)  LYQDEIKTFTMWYNQELGPKKLSFSRDRAVENYFWALGIMHFEPELATGRLLLAKLLAYVLVWDDMYDAYGTFDELRLFTDVIERWDLESVDHLPEYMRVCLSSYSNFMREVEDELIKNE
FhTPS8 (Ambiance)    LYQDEIKTFTMWYNQELGPKKLSFSRDRAVENYFWALGIMHFEPELATGRLLLAKLLAYVLVWDDMYDAYGTFDELRLFTDVIERWDLESVDHLPEYMRVCLSSYSNFMREVEDELIKNE
                     ************************************************************************************************************************

FhTPS8 (Red River®)  RDQLKPYVAEMMKYIIDGFFQEAKWLNEGYIPTMEEYLANGLKTGGQTTLNGFSLLFMSEDKVTKDTLEWVLSMPNILKASTLIGRLLNDIKTTKLEHERMHVASSIQIYMNEAGVTEAM
FhTPS8 (Ambiance)    RDQLKPYVAEMMKYIIDGFFQEAKWLNEGYIPTMEEYLANGLKTGGQTTLNGFSLLFMSEDKVTKDTLEWVLSMPNILKASTLIGRLLNDIKTTKLEHERMHVASSIQIYMNEAGVTEAM
                     ************************************************************************************************************************

FhTPS8 (Red River®)  AIAKLNGMVADFWKDINKELLDALPFQKDFNTLTLNFARTLEVLYKHEDAFTHGSIQREQIDLMLVWPIQN
FhTPS8 (Ambiance)    AIAKLNRMVADFWKDINKELLDALPFQKDFNTLTLNFARTPEVLYKHEDAFTHGSIQREQIDLMLVWPIQN
                     ****** ********************************* ******************************
```

Fig. S8. Alignment of deduced amino acid sequences of FhTPS8 in Red River® and Ambiance.

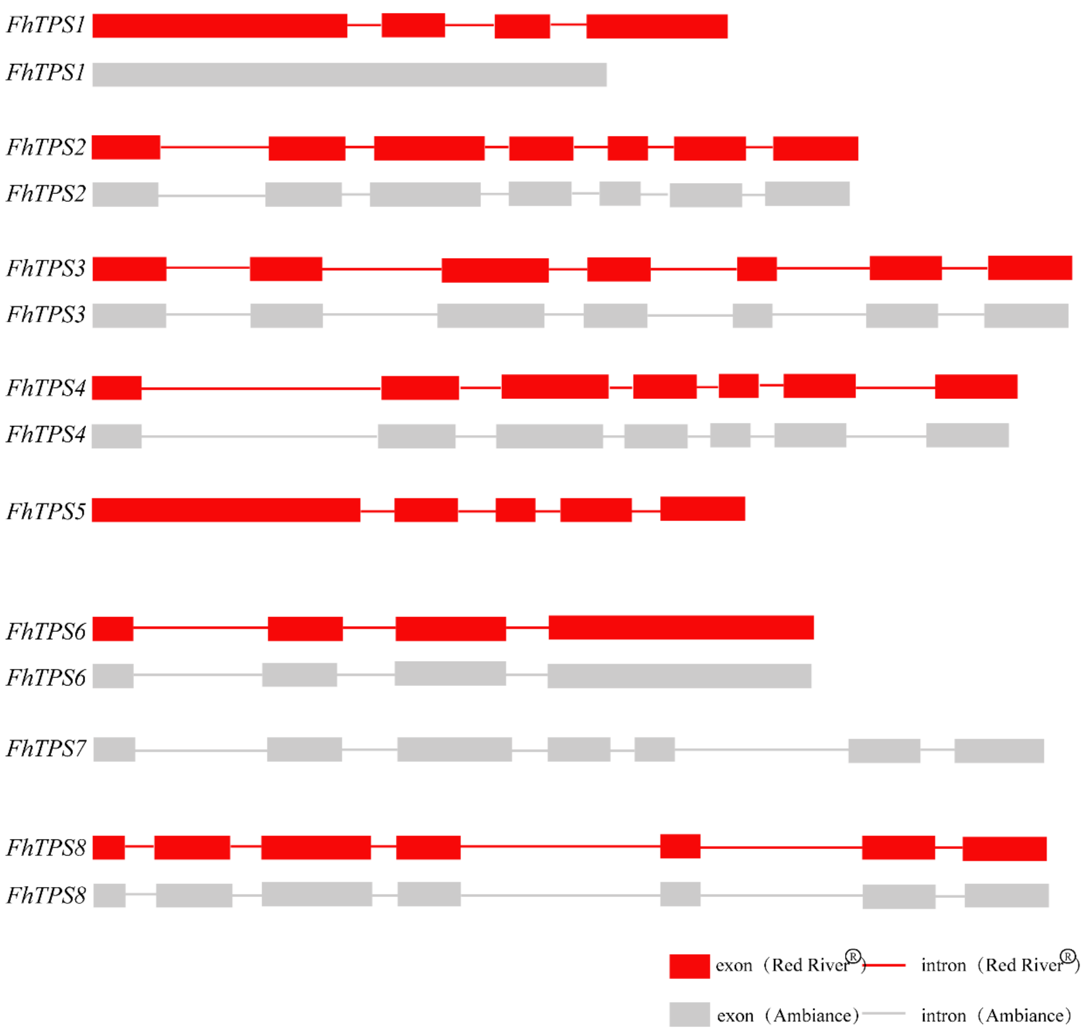

Fig. S9. Genomic structures of the *FhTPS* genes for two cultivars of *Freesia hybrida* (Red River® and Ambiance).

Genomic organization of *FhTPS* genes. The frames and solid lines represented exons and introns, respectively.

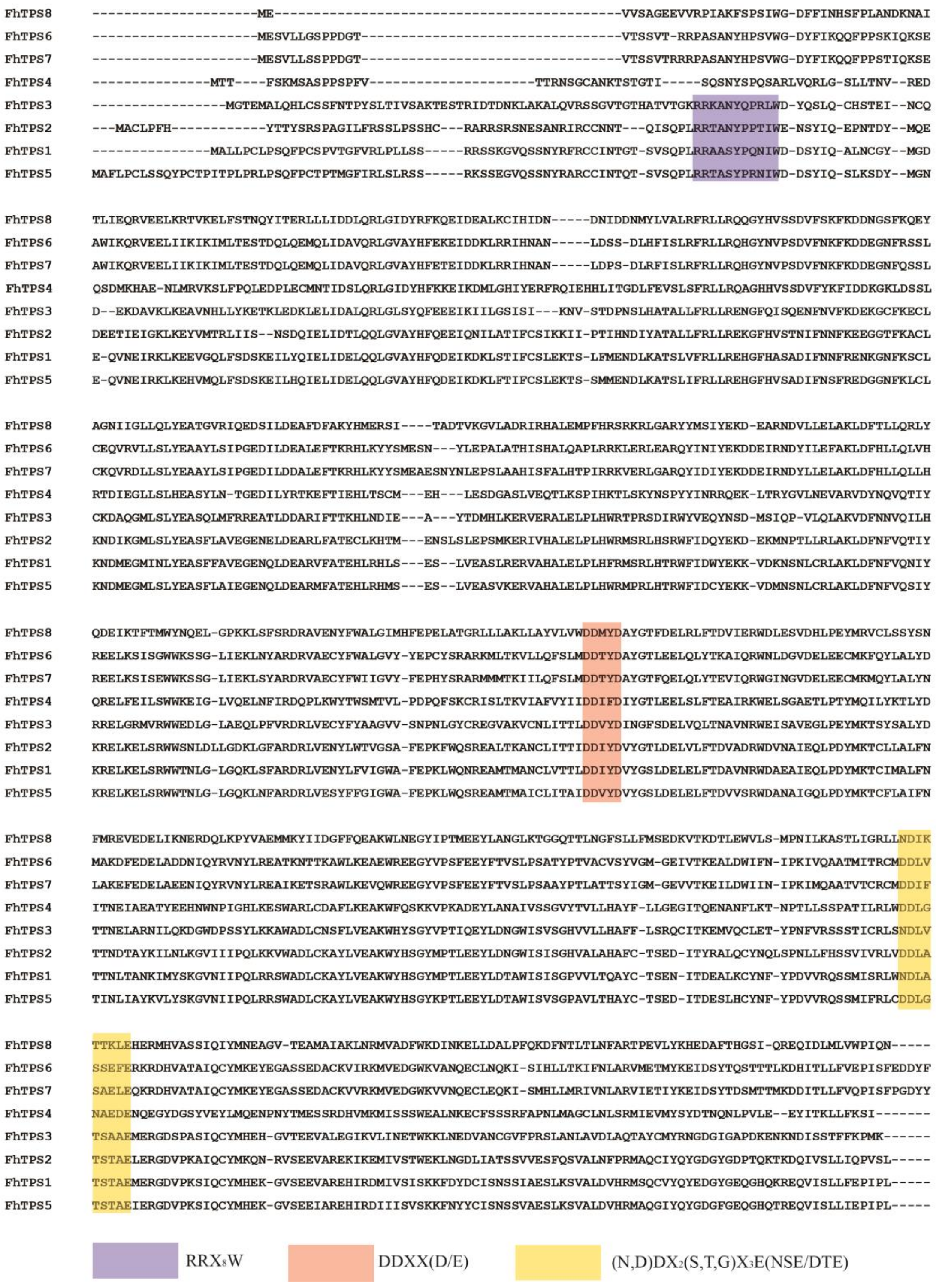

Fig. S10. Conserved residues analysis and subcellular localization of FhTPS proteins in two cultivars of *Freesia hybrida*

Full-length protein sequence alignment of Freesia TPS proteins (FhTPS1-FhTPS8). Functionally important conserved residues (RRX_8W, DDXXD and DDXXSXXXE/ NDXXTXXXE) are highlighted with a colored background.

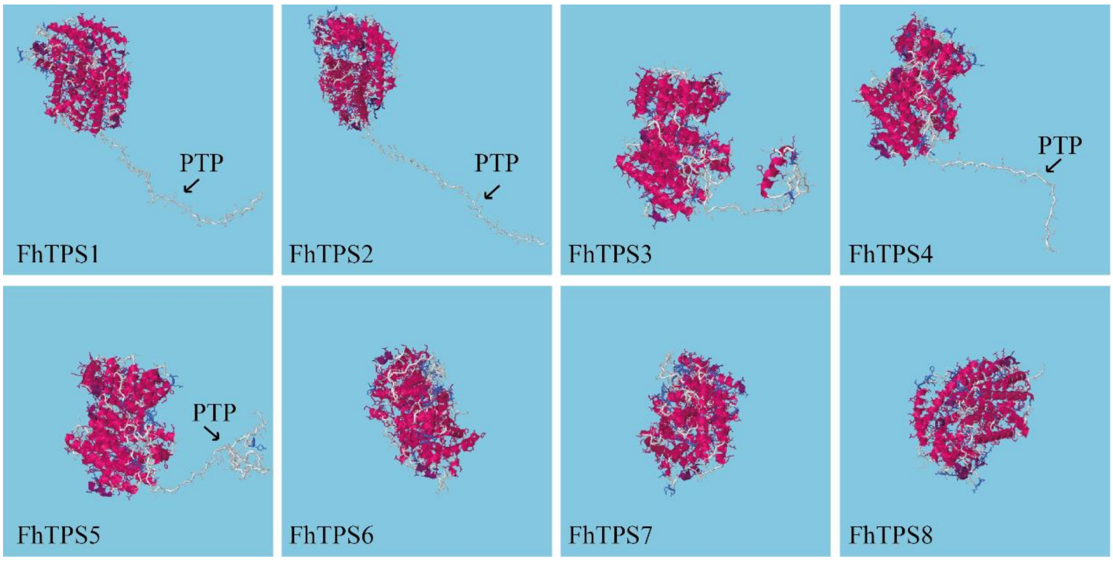

Fig. S11. Three-dimensional model of the structure model of FhTPSs.

The globular protein is shown in pink and the chloroplast transit peptide (PTP) in white.

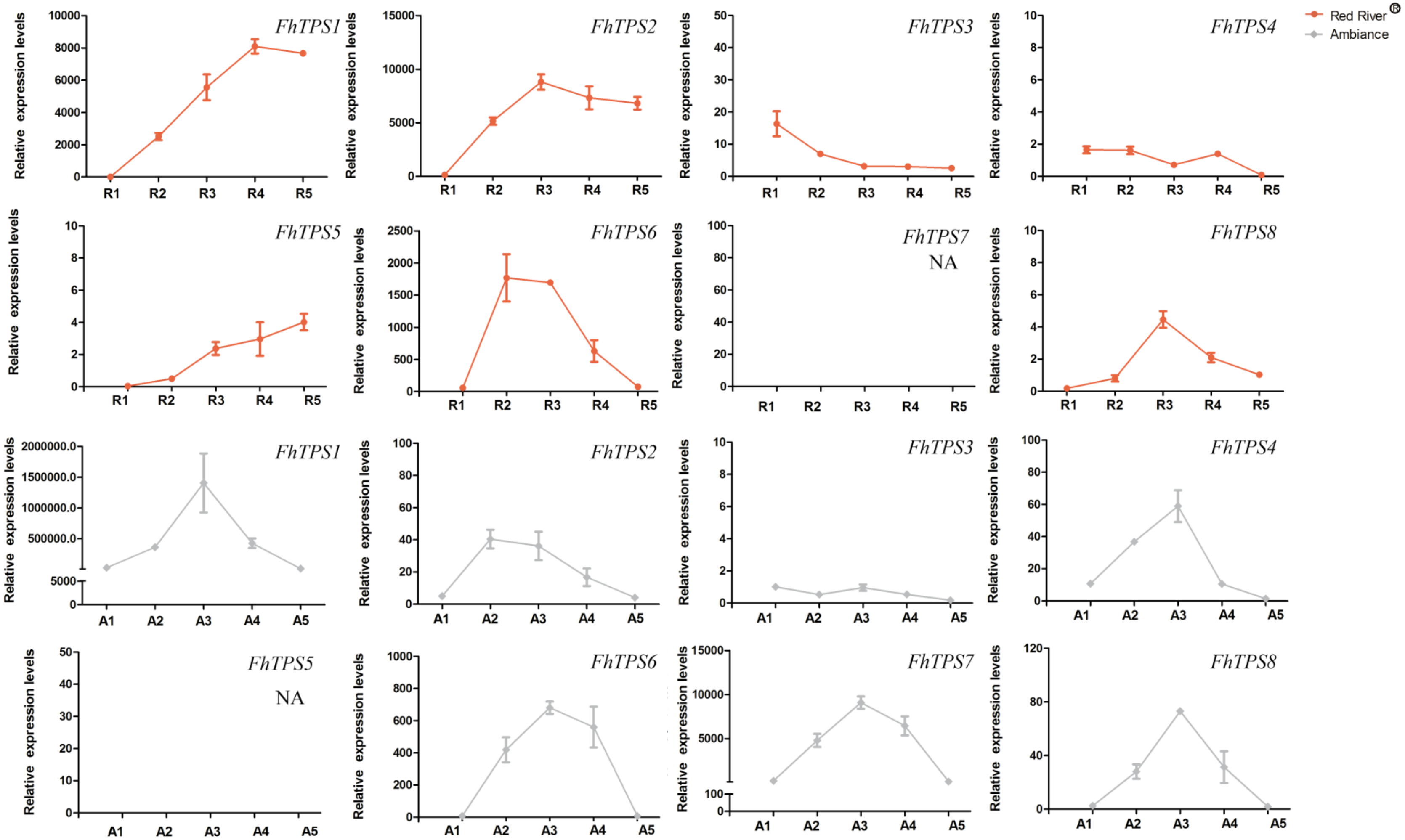

Fig. S12. Expression patterns of *FhTPS* genes in five developmental stages of Red River® and Ambiance

Real-time PCR gene expression analysis of *FhTPS* genes at five development stages of Red River® and Ambiance. Relative gene expression levels were calculated with formula $2^{-\Delta\Delta C_T}$. Gene expression levels of *FhTPS* genes in Red River® and Ambiance are represented by red and gray lines, respectively. NA indicates no gene expression. The flower developmental stages and tissues were defined as in previous studies (Li et al., 2016; Sun et al., 2016; Sun et al., 2016). All results are presented as means ± SD of triplicate experiments.

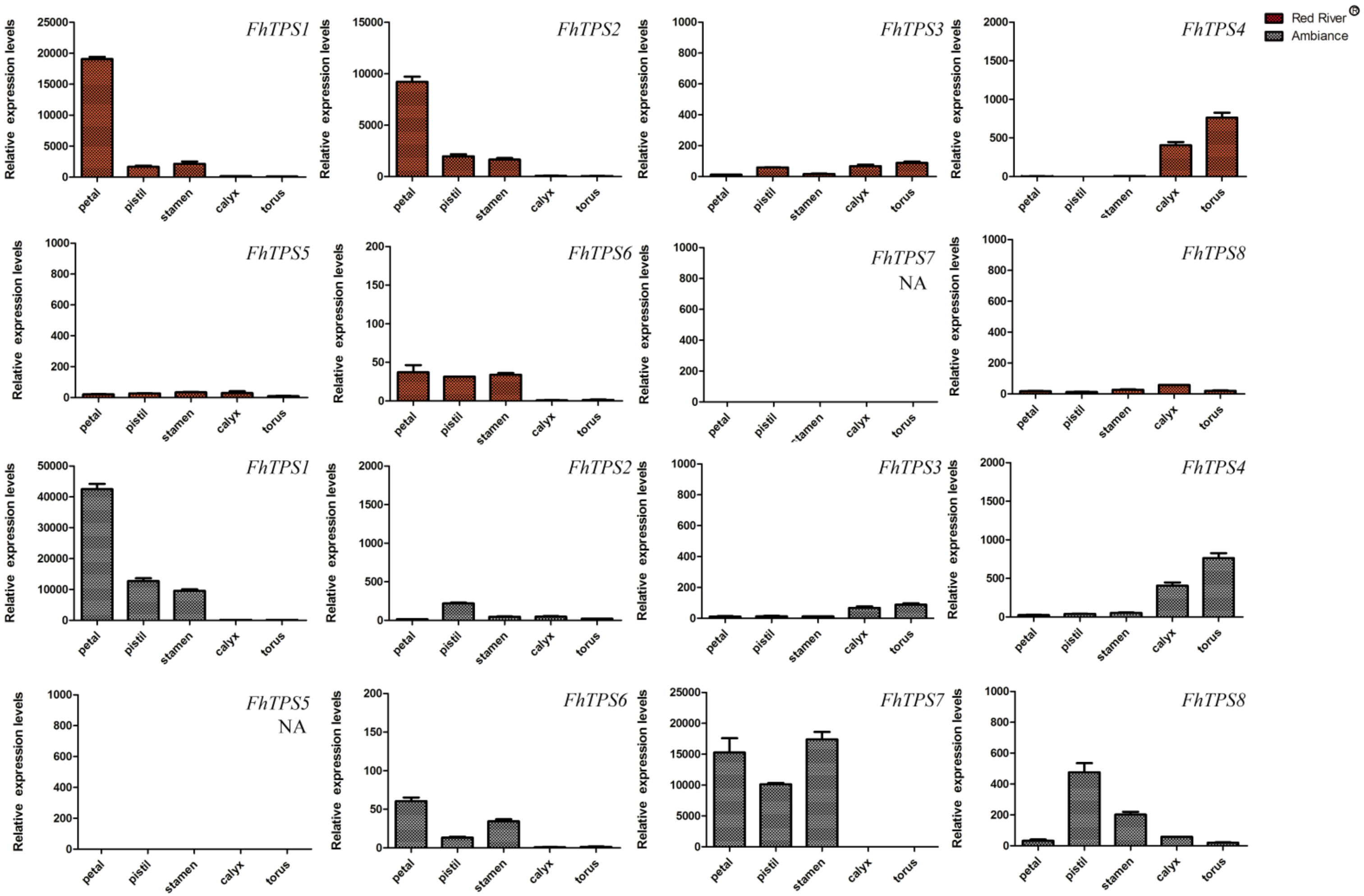

Fig. S13. Expression patterns of *FhTPS* genes in five flower tissues of Red River® and Ambiance

Real-time PCR gene expression analysis of *FhTPS* genes in five flower tissues of Red River® and Ambiance. Relative gene expression levels were calculated with formula $2^{-\Delta\Delta C_T}$. NA indicates no gene expression. Gene expression levels of *FhTPS* genes in Red River® and Ambiance are represented by red and gray frames, respectively. All results are presented as means ± SD of triplicate experiments.

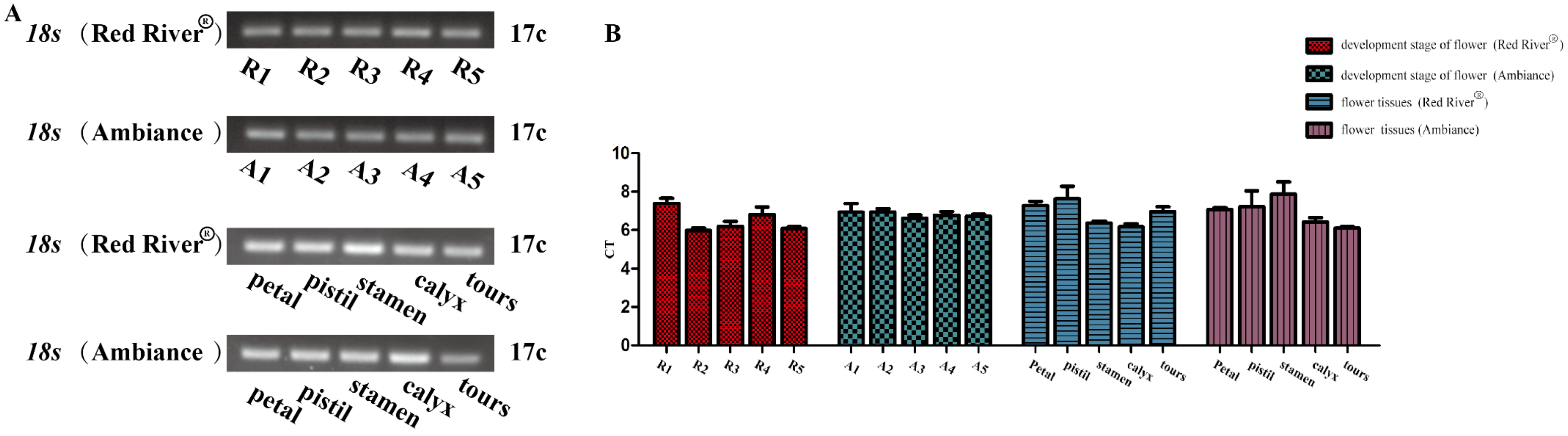

Fig. S14. Stability analysis of 18S rRNA in two cultivars of *Freesia hybrida*.

(A) The expression of 18S rRNA was analyzed at five developmental stages and in five flower tissues of two cultivars of *Freesia hybrida* by semi-quantitative PCR. 17C represented 17 PCR cycle. (B) The expression of 18S rRNA was analyzed at five developmental stages and in five flower tissues of two cultivars of *Freesia hybrida* by real-time PCR. The number in Y-axis represented the CT value of 18S rRNA.

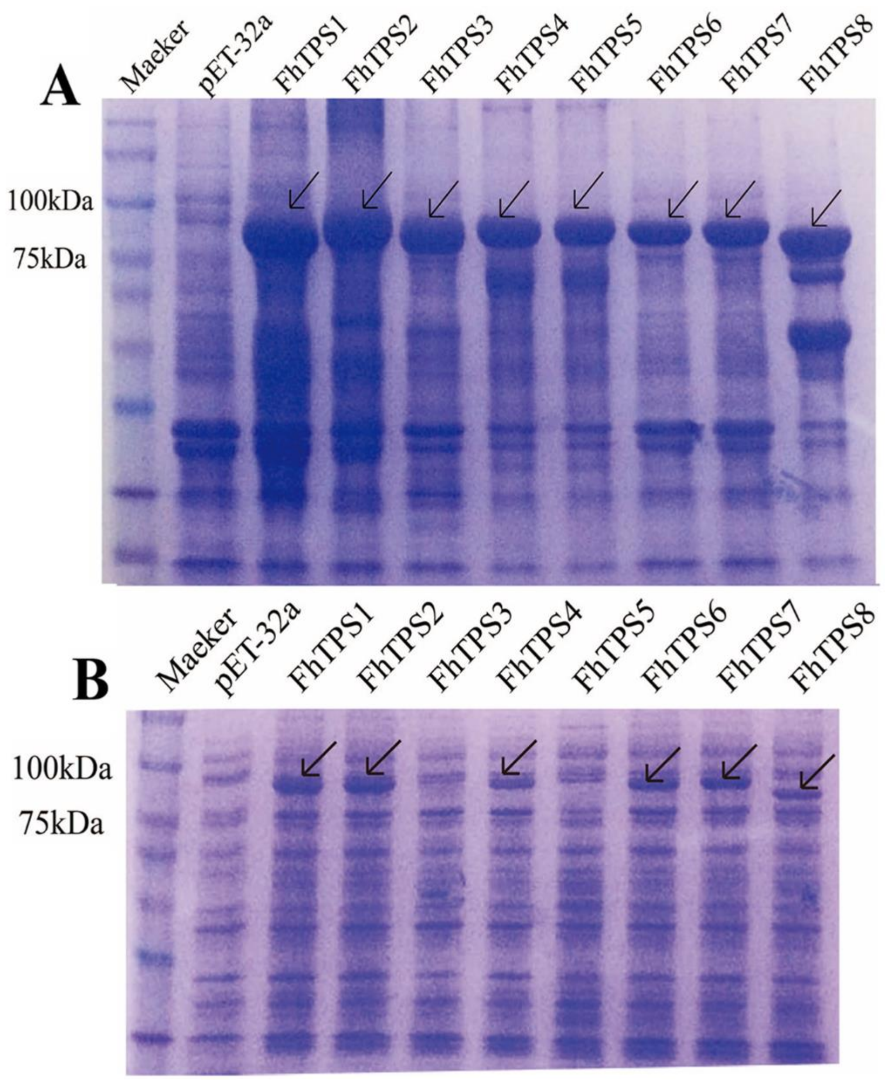

Fig. S15. Detection of FhTPS proteins in *E. coli* strain BL21 (DE3)

(A) SDS-PAGE gel showing recombinant 6His-tagged FhTPSs (FhTPS1-FhTPS8) proteins in the form of inclusion body protein. (A) SDS-PAGE gel showing recombinant 6His-tagged FhTPSs (FhTPS1-FhTPS8) proteins in the form of soluble protein. The position corresponding to the recombinant proteins are indicated by an arrow.

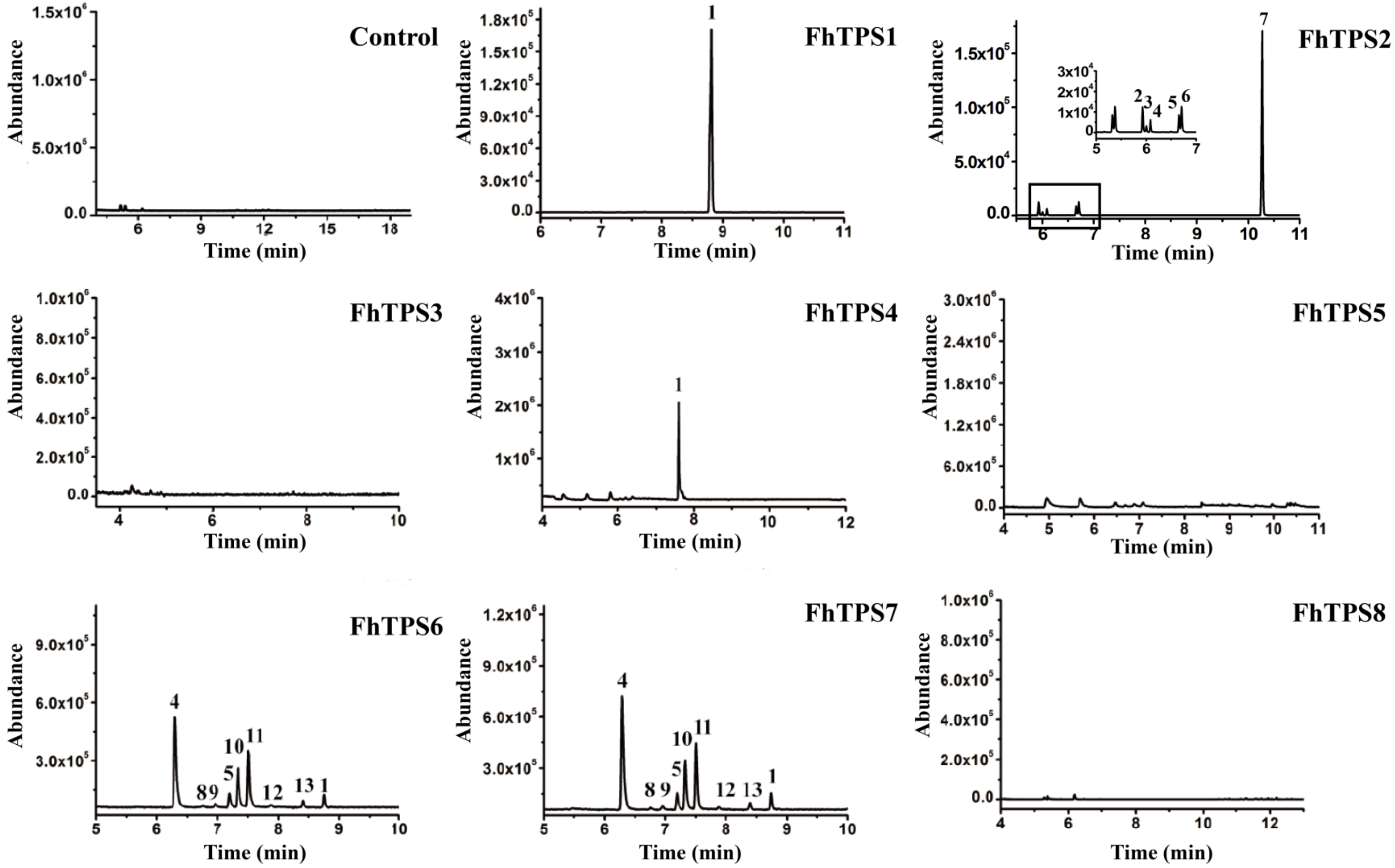

Control
FhTPS1
FhTPS2
FhTPS3
FhTPS4
FhTPS5
FhTPS6
FhTPS7
FhTPS8
Abundance
Time (min)

Fig. S16. *In vitro* enzymatic activity analysis of FhTPS proteins using GPP as substrate

Products catalyzed by FhTPS proteins (FhTPS1-FhTPS8) using GPP as substrate were subjected to GC-MS. Peaks marked with numbers were identified as monoterpenes by comparing mass spectra with the NIST 2008 mass spectra library. Each experiment was performed in three independent repeats to confirm the accuracy of the FhTPS products. The X axis represents the retention time of the peak outflow, and the Y axis represents the integral area of chromatographic peak. The enzymatic products are as follows: 1, Linalool; 2, Bicyclo [3.1.0] Thujene; 3, α-Pinene; 4, Myrcene; 5, D-Limonene; 6, 1,8 cineole; 7, α-Terpineol; 8, Thujene; 9, Isoterpinolene; 10, cis-Ocimene; 11, trans-Ocimene; 12, Terpinene; 13, Terpinolene.

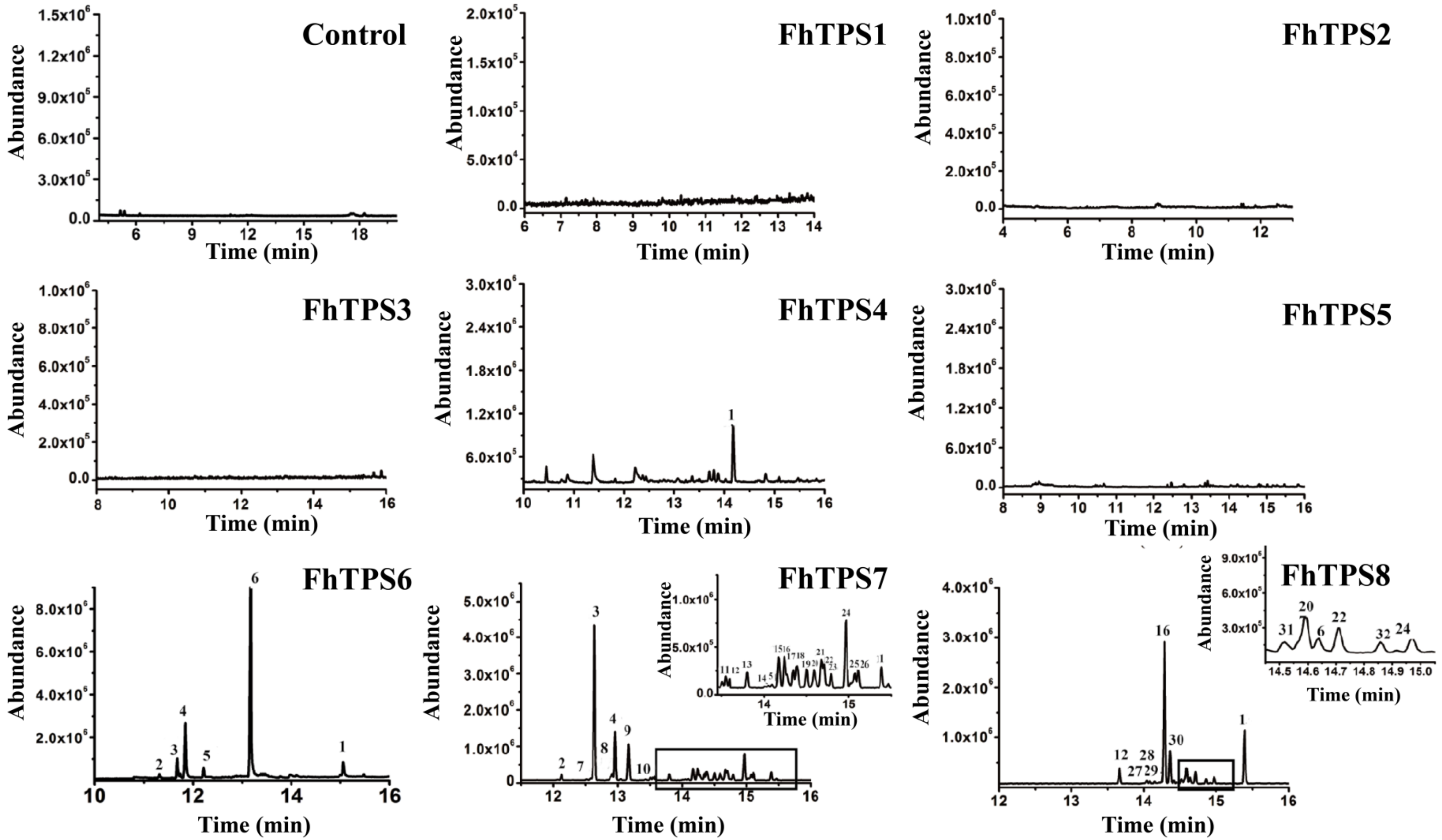
Control
FhTPS1
FhTPS2
FhTPS3
FhTPS4
FhTPS5
FhTPS6
FhTPS7
FhTPS8
Abundance
Time (min)

Fig. S17. ***In vitro*** **enzymatic activity analysis of FhTPS proteins using FPP as substrate**

Products catalyzed by FhTPS proteins (FhTPS1-FhTPS8) using FPP as substrate were subjected to GC-MS. Peaks marked with numbers were identified as sesquiterpenes by comparing mass spectra with the NIST 2008 mass spectra library. Each experiment was performed in three independent repeats to confirm the accuracy of the FhTPS products. The X axis represents the retention time of the peak outflow, and the Y axis represents the integrated area of the chromatographic peak. The enzymatic products are as follows: 1, Nerolidol; 2, α-Cubebene; 3, Copaene; 4, Elemene; 5, Caryophyllene; 6, Selinene; 7, Cycloisosativene; 8, Epi-bicyclosesquiphellandrene; 9, β-Maaliene; 10, β- Caryophyllene; 11, α-Guaiene; 12, Farnesene; 13, γ-Maaliene; 14, Himachalene; 15, Sativene; 16, α-Gurjunene; 17, γ-Cadinene; 18, γ-Muurolene; 19, γ-Gurjunene; 20, Germacrene; 21, α- Muurolene; 22, Guaia-1(10), 11-diene; 23, Eudesmene; 24, Cadina-3, 9-diene; 25, 1R, 3Z, 9S-2, 6, 10, 10-Tetramethylbicyclo [7.2.0] undeca-2, 6-diene; 26, Naphthalene 1, 2, 3, 4, 4a, 7-hexahydro-1, 6-dimethy-4-(1-methylethl); 27, Isoledene; 28, unknown; 29, Acoradien; 30, Chamigrene; 31, Zingberene; 32, Sesquiphellandrene.

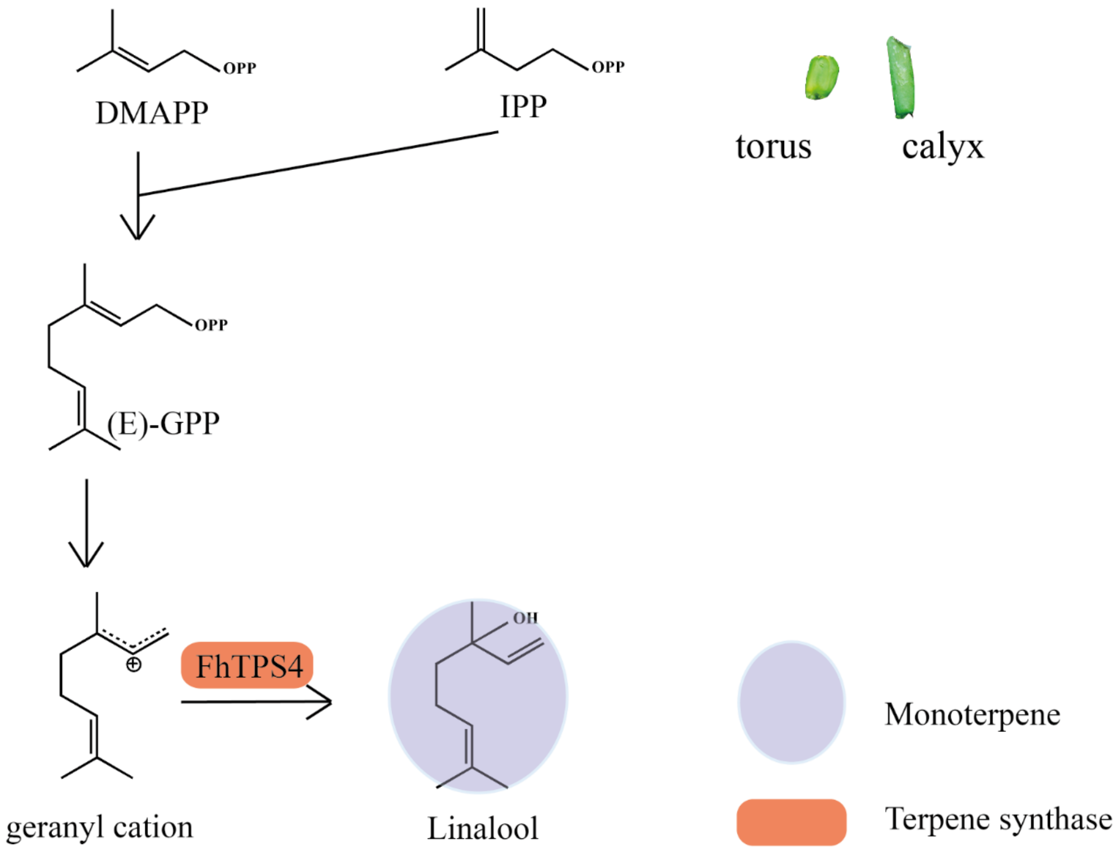

Fig. S18. Proposed model of terpene biosynthesis in torus and calyx of both *Freesia* cultivars.

FhTPS4 was specifically associated with linalool biosynthesis in these two flower tissues, and nerolidol, which was found in the *in vitro* enzymatic products catalyzed by FhTPS4, was not detected.

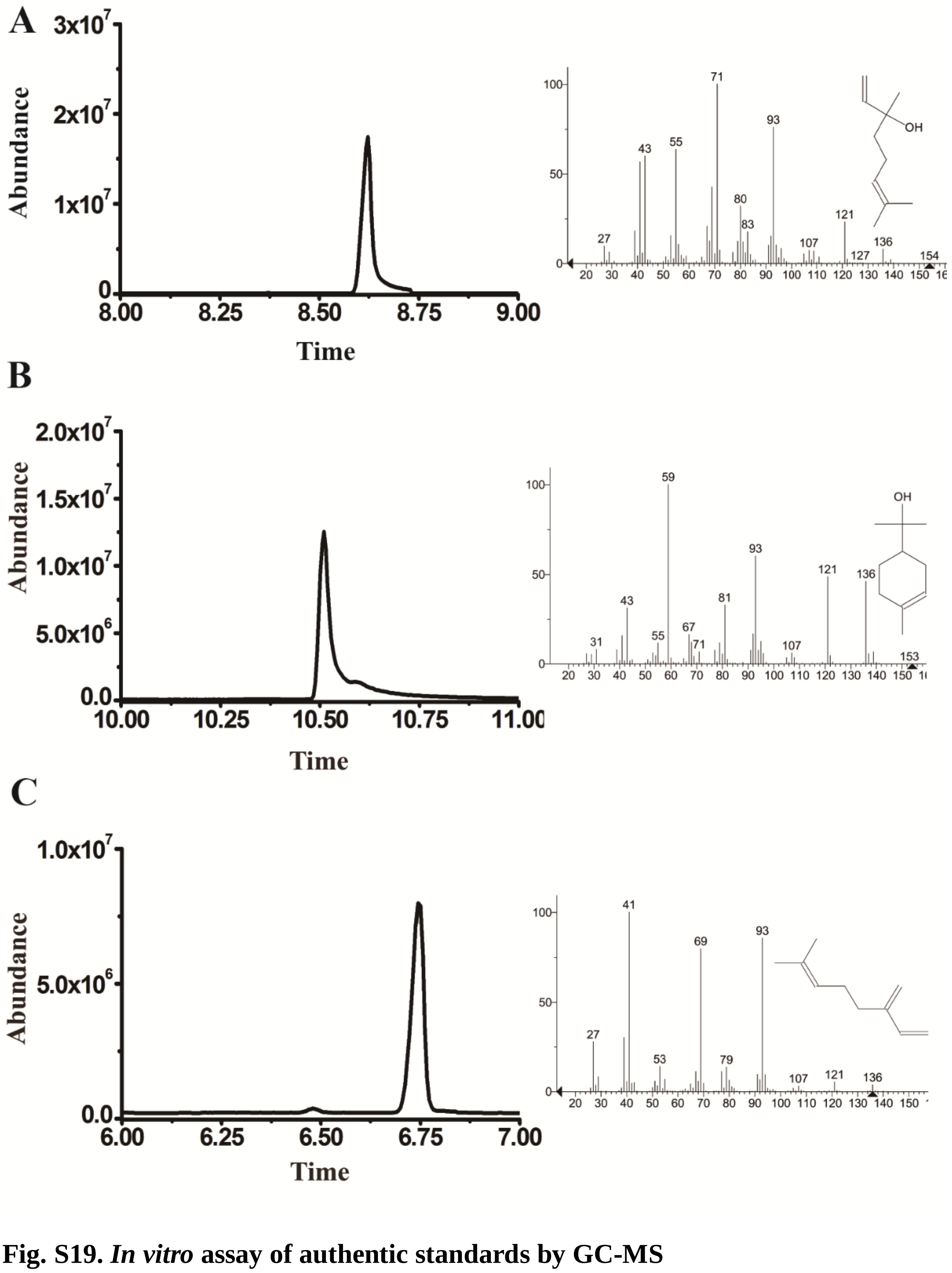

Fig. S19. *In vitro* assay of authentic standards by GC-MS

(A) Linalool. (B) α-Terpilenol. C. Myrcene. Mass spectra for each authentic standard are shown on the right.
